# Discovery of a Distinct Superfamily of Kunitz-Type Toxin (KTT) from Tarantulas

**DOI:** 10.1371/journal.pone.0003414

**Published:** 2008-10-15

**Authors:** Chun-Hua Yuan, Quan-Yuan He, Kuan Peng, Jian-Bo Diao, Li-Ping Jiang, Xing Tang, Song-Ping Liang

**Affiliations:** The Key Laboratory for Protein Chemistry and Developmental Biology of Ministry of Education, College of Life Sciences, Hunan Normal University, Changsha, People's Republic of China; University of the Western Cape, South Africa

## Abstract

**Background:**

Kuntiz-type toxins (KTTs) have been found in the venom of animals such as snake, cone snail and sea anemone. The main ancestral function of Kunitz-type proteins was the inhibition of a diverse array of serine proteases, while toxic activities (such as ion-channel blocking) were developed under a variety of Darwinian selection pressures. How new functions were grafted onto an old protein scaffold and what effect Darwinian selection pressures had on KTT evolution remains a puzzle.

**Principal Findings:**

Here we report the presence of a new superfamily of KTTs in spiders (Tarantulas: Ornithoctonus huwena and Ornithoctonus hainana), which share low sequence similarity to known KTTs and is clustered in a distinct clade in the phylogenetic tree of KTT evolution. The representative molecule of spider KTTs, HWTX-XI, purified from the venom of O. huwena, is a bi-functional protein which is a very potent trypsin inhibitor (about 30-fold more strong than BPTI) as well as a weak Kv1.1 potassium channel blocker. Structural analysis of HWTX-XI in 3-D by NMR together with comparative function analysis of 18 expressed mutants of this toxin revealed two separate sites, corresponding to these two activities, located on the two ends of the cone-shape molecule of HWTX-XI. Comparison of non-synonymous/synonymous mutation ratios (ω) for each site in spider and snake KTTs, as well as PBTI like body Kunitz proteins revealed high Darwinian selection pressure on the binding sites for Kv channels and serine proteases in snake, while only on the proteases in spider and none detected in body proteins, suggesting different rates and patterns of evolution among them. The results also revealed a series of key events in the history of spider KTT evolution, including the formation of a novel KTT family (named sub-Kuntiz-type toxins) derived from the ancestral native KTTs with the loss of the second disulfide bridge accompanied by several dramatic sequence modifications.

**Conclusions/Significance:**

These finding illustrate that the two activity sites of Kunitz-type toxins are functionally and evolutionally independent and provide new insights into effects of Darwinian selection pressures on KTT evolution, and mechanisms by which new functions can be grafted onto old protein scaffolds.

## Introduction

Developing venoms to kill or paralyze prey provides an important means for venomous animals to interact with their environment. Under great Darwinian selection pressure, venomous animals strive to construct more efficient toxins so as to be evolutionarily successful. It was hypothesized that the evolution of the animal venom proteome comprises a series of key events including recruitment of an existing ancestor gene, gene duplications and focal hypermutation [1]–[3]. This process has been of tremendous research interest and considerable debate.

The Kunitz type motif usually has a peptide chain of around 60 amino acid residues and is stabilized by three disulphide bridges with the bonding pattern of 1–6,2–4, 3–5. This motif was first seen in the bovine pancreatic trypsin inhibitor (BPTI)-like proteinase inhibitors, which are exceptionally strong inhibitors of serine proteinases (also known as S1A proteases) such as trypsin and chymotrypsin. The structure-function relationships of BPTI-like proteinase inhibitors have been extensively studied. The 3D-structure of BPTI, determined by both crystallography and NMR, reveals an α/β/α structural motif [4], [5] The structure-function relationship analysis of BPTI has shown that a solvent exposed loop (from residue 8 to19) is highly complementary to the enzyme active site (S1 pocket), wherein a P1 residue (Lys15 in BPTI) penetrates deeply and interacts with Asp 189 at the bottom of the SI pocket[6]. Kunitz type proteinase inhibitors may be a kind of “old” molecules because that they are ubiquitous in numerous organisms, including plants, animals and microbes.

The first Kunitz type toxin (KTT) in animal venom (Swissprot No: P00979) was isolated from snake in 1974. After that, many KTTs were found in various venomous animals, including snakes, lizards, cattle ticks, cone snails and sea anemones [7]–[10]. Besides with the original function (serine protease inhibition), some of them have the ability to block ion channels, especially the voltage-gated potassium channels, which are essential for regulation of various physiological processes such as blood coagulation, fibrinolysis, host defense and action potential transduction. Therefore, these are of potential value not only for evolutionary research, but also for drug design. The 3D structures of dendrotoxin-K (DTX-K) and dendrotoxin 1 (DTX-1), two typical snake KTTs and the potent potassium channel blockers without inhibitory activities for proteases, have been determined by NMR[11], [12]. The molecular architectures of DTXs are essentially identical to the structure of BPTI. The structure-function relationship studies of DTXs reveals that the important amino acid residues responsible for the K^+^ channel binding are located in the N terminus and β-turn regions[13].

Although it has been hypothesized that the old Kunitz type scaffold is recruited by the venomous animals to produce KTTs in their venom during the evolution [2], some fundamental questions–for example, whether the dual functions are tightly bound or not; how a new neurotoxic function can be grafted onto an old proteinase scaffold; and what the effect of Darwinian selection pressure on the evolution of the KTTs is– remain unanswered, possibly because there is limited sequence and three-dimensional structural data for the KTTs. For example, there is only one cone snail KTT, and 6 of 9 cattle tick KTT peptides are fragments according to SwissProt version 51.4, and the lack of nucleic acid sequences (in sea anemone). Additionally, in most cases, evolutionary analyses of KTTs are restricted to one taxonomic group, and systematic comparisons between them are rare[14], [15]. Here, we report the first superfamily of KTTs from spiders and discuss the structure and functions of a representative toxin (HWTX-XI) to provide a global view of KTTs evolution across taxonomic groups, as well as clues toward the answer to the foregoing questions.

## Results

### Purification and amino acid sequence of HWTX-XI

**Figure 1:**
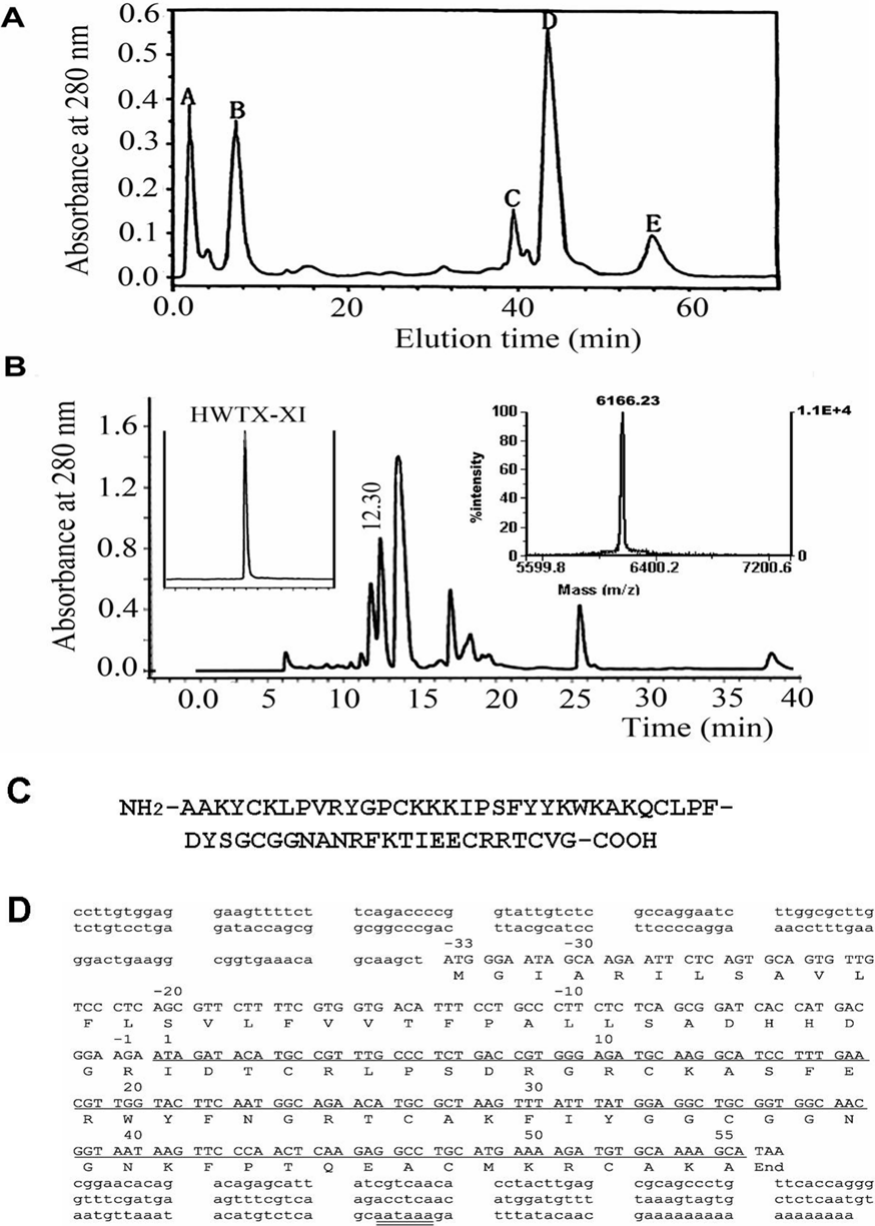
Purification and amino acid sequence of HWTX-XI. (A) Ion-exchange HPLC chromatograph of crude Ornithoctonus huwena venom. The column (Waters Protein-Pak CM, 10 mm×100 mm) was equilibrated with 0.1 M sodium phosphate buffer, pH 6.8, the crude venom (10 mg in 2 ml) was loaded and eluted at a flow rate of 3 mL/min, using a gradient of 0–81% buffer B (1 M NaCl, 0.1 M sodium phosphate buffer, pH 6.8 ) over 60 min. Then the peak E was applied to Vydac C18 reverse phase column (4.6 mm×250 mm) equilibrated with 0.1% trifluoroacetic acid (B), using a gradient of 20–40% buffer C (0.1% trifluoroacetic acid in acetonitrile) over 45 min. The fraction of retention time 12.30 min containing HWTX-XI was further purified by Vydac C18 rpHPLC as shown in the left inset. The molecular mass of HWTX-XI was determined by MALDI-TOF mass spectrometry (right inset of B). (C) Amino acid sequence of HWTX-XI determined by Edman degradation. (D) The oligonucleotide sequence of HWTX-XI cDNA. The cDNA encoding the mature peptide are underlined. The polyadenylations signal, AATAAA, is double underlined.

HWTX-XI was purified using ion-exchange HPLC combined with reverse-phase HPLC (Fig. 1A,B) from the venom of *Ornithoctonus huwena*. The amino acid sequence of HWTX-XI, as shown in Fig. 1C, was determined by Edman degradation. It is composed of 55 amino acid residues, including six cysteines. Its exact molecular mass is 6166.23 Da, as determined by MALDI-TOF mass spectrometry. Since the mass calculated based on sequence analysis is 6172.2, the difference of 6 mass units indicates that all of the six Cys residues are involved in 3 disulfide bonds. The amino acid sequence of HWTX-XI was also confirmed from its cDNA sequence obtained using 3′-and 5′-RACE methods (Fig. 1D). The open reading frame encoded a potential signal peptide of 23 residues, a mature peptide of 55 residues and a 10 residue intervening pro-protein region. The deduced amino acid sequence was consistent with the sequence determined by Edman degradation. The amino acid sequence follows the native Kunitz motif, as characterized by the sequence similarity and the location of the six systeine residues, which are similar to those seen in BPTI (a trypsin inhibitor from bovine pancreas) and DTX-K (a K^+^ channel blocker from snake *Dentroaspis polylepis*) [16], [17]. However, there are some difference between the sequences of HWTX-XI and other Kunitz type molecules (See below).

### Biological Functions of HWTX-XI

**Figure 2:**
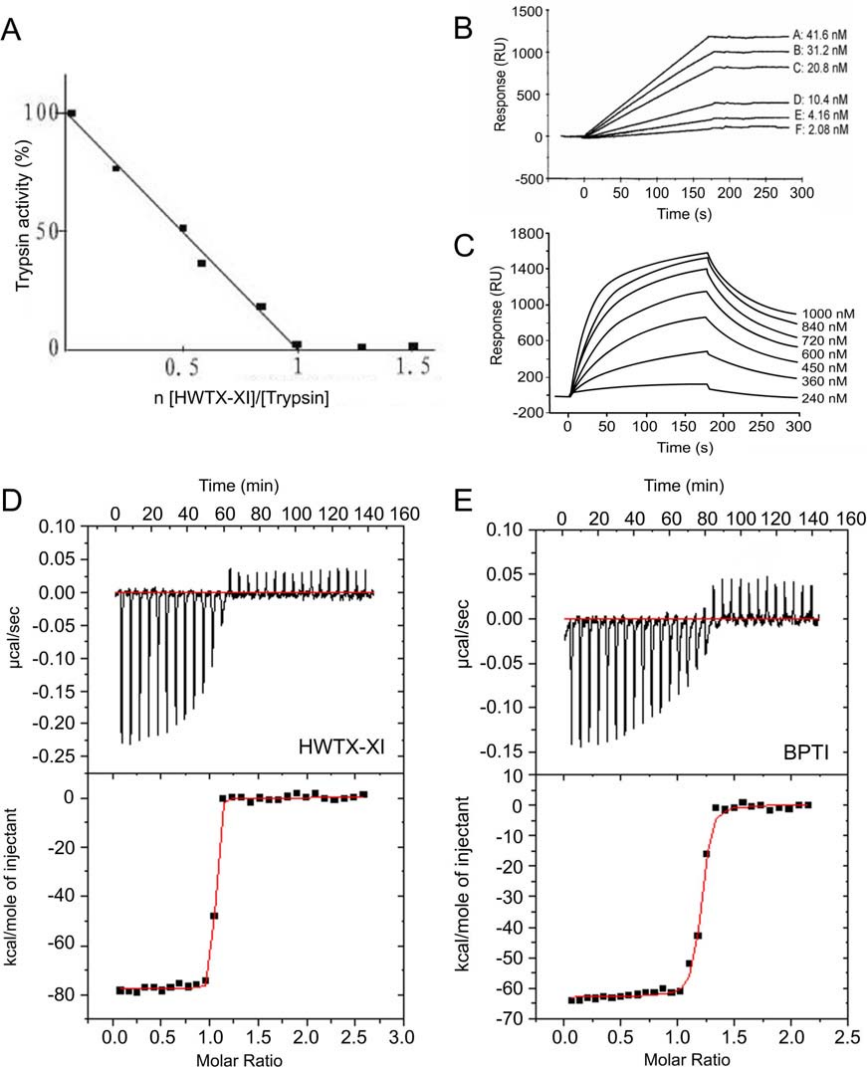
The inhibitory activities of HWTX-XI to proteinases. (A) The inhibition stoichiometry of HWTX-XI to trypsin. Trypsin assay was performed in 100 mM Tris-HCl (pH 8.0), containing 20 mM CaCl_2_ and 0.05% triton X-100. Trypsin was incubated with various amount of HWTX-XI for 10 min with the substrate Benzoyl-L-arginine-p-nitroanilide (BAPNA) at a final conaentration of 0.4 mM. The protease activity was monitored at 405 nm. (B) BIAcore analysis of immobilized HWTX-XI binding to trypsin (2.08–41.6 nM) with a BIAcore X instrument (BIAcore AB, Uppsala, Sweden). HWTX-XI (11.5 µM,desolved in 10 mM sodium acetate,pH 5.5)was coupled to a carboxymethylated dextran CM5 sensor chip. The binding assay was performed with a constant flow rate of 20 µl/min at 25°C. (C) BIAcore analysis of immobilized HWTX-XI binding to α-chymotrypsin (240 nM to 1000 nM). (D) and (E) Isothermal titration calorimetry data of HWTX-XI (D) and BPTI (E) titrated with trypsin. Experiments were conducted with a VP-ITC system at 25°C with stirring at 300 rpm. Top, raw data (cal/s vs. time) showing heat release upon injection of 0.01 mM trypsin into a 1.4-ml cell containing 0.001 mM HWTX-XI (D) or BPTI (E). Bottom, integration of the raw data yields the kcal/mol vs. molar ratio.

Native HWTX-XI was investigated in order to determine its biological activities. The trypsin inhibitory activity of HWTX-XI was determined using the substrate Benzoyl-L-arginine-p-nitroanilide (BAPNA) as shown in Fig. 2A, which shows that HWTX-XI inhibits trypsin stoichiometrically in a ratio 1∶1.Fig. 2B and 2C show the binding of HWTX-XI to trypsin, and α-chymotrypsin, respectively, as measured by surface plasmon resonance using a BIAcore X instrument. The results shown in Fig. 2B indicated that at very low concentration, trypsin binds strongly to HWTX-XI immobilized on the sensor chip: there is almost no dissociation during wash phase. By comparison, the binding of α-chymotrypsin to HWTX-XI is relatively lower. The dissociation constant was measured using isothermal titration calorimetry (ITC).Fig. 2D and 2E show the titration curves used to determine the thermodynamic parameters for the binding of HWTX-XI and BPTI to trypsin. Analysis of the data revealed an 1∶1 stoichiometry of the two binding partners, and a *Kd* value of 2.3×10^−10^ M for HWTX-XI binding to trypsin. By compassion, the *Kd* for binding of BPTI to trypsin was determined to be 6.57×10^−9^ M. This result shows that HWTX-XI has 29-fold higher inhibition potential compared with BPTI, a well-known strong trypsin inhibitor.

**Figure 3:**
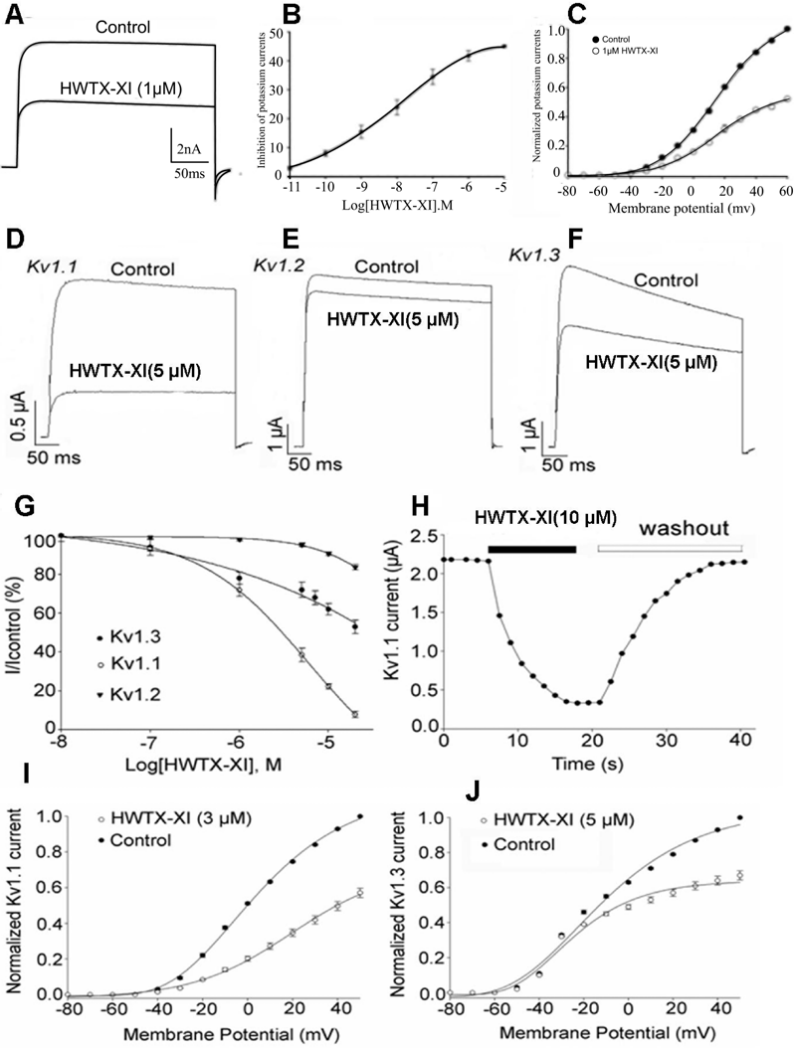
Effects of HWTX-XI on Kv channels. (A) 1 µM HWTX-XI evidently reduced the control potassium currents amplitude in rat DRG neurons by 41.7±1.8% (n = 5). (B) Concentration-response relationship for HWTX-XI inhibition of potassium currents expressed on rat DRG neurons. Each data point (mean±S.E.) arises from 5–7 cells. The solid line through the data is a fit of *I*/*I_max_* = 1/[1+exp(*C*–*IC_50_*)/*Κ*]. (C) Effect of HWTX-XI on steady-state current-voltage relationship of potassium channels on rat DRG neurons. DRG cells were held at −80 mV and stepped to test potentials of −80 to +60 mV (mean±SD, *n* = 4). (D–F) 1 µM HWTX-XI evidently reduced the control potassium currents (Kv1.1, D; Kv1.2, E; Kv1.3, F) amplitude in rat DRG neurons by 41.7±1.8% (n = 5). (*G*) Concentration-response relationship for HWTX-XI inhibition of potassium currents expressed on rat DRG neurons. Each data point (mean±S.E.) arises from 5–7 cells. The solid line through the data is a fit of *I*/*I_max_* = 1/[1+exp(*C*−*IC_50_*)/*Κ*]. (H) At a concentration of 10 µM, HWTX-XI produced a rapid (τ≈12±2 s for steady-state inhibition, n = 5) inhibition which is readily reversible with the time constant of 21±3 s upon removal of the toxin. (I–J) The current-voltage curves of Kv1.1 (I) and Kv1.3(J) currents activated by 3 µM and 5 µM HWTX-XI respectively.

The toxin also can inhibit potassium channels expressed in rat dorsal root ganglion neurons (Fig. 3A–C). As shown in Fig. 3A, 1 µM HWTX-XI reduced the amplitude of control potassium currents maximally by 41.7±1.8% (n = 5). The inhibition of HWTX-XI was concentration-dependent with an IC_50_ value of 11.6 nM (Fig. 3B). Current-voltage curves showed that potassium currents were activated at around −30 mV. From the current-voltage relationship, it was determined that HWTX-XI produced a distinct inhibition of potassium currents at all test pulses, and that the inhibition was voltage-dependent (Fig. 3C).

We also tested the effects of HWTX-XI on K^+^ channel subtypes expressed in *X. laevis* oocytes using the two-microelectrode voltage-clamp technique (Fig. 3 D–J). These results indicated that HWTX-XI is relatively specific for Kv1.1 channels where blockage was 78±7% (n = 5) at a concentration of 5 µM (Fig. 3D), but just less for Kv1.2 and Kv1.3 channels (10±2% and 28±3% inhibition, respectively, at the concentration of 5 µM, n = 5), (Fig. 3E and 3F). Fig. 3G shows the concentration-response relationship for HWTX-XI inhibition of Kv1.1, Kv1.2 and Kv1.3 currents at 20 mV. Every data point (mean±S.E.) coming from 5–8 cells shows current relative to control. These data points were fitted according to the Boltzmann equation; % inhibition = 100/[1+exp(C−IC_50_)/*k*], where IC_50_ is the concentration of toxin at half-maximal inhibition, *k* is the slope factor, and C is the toxin concentration. At a concentration of 10 µM, HWTX-XI produced a rapid inhibition (τ≈12±2 s for steady-state inhibition, n = 5), which was readily reversible, with the time constant of 21±3 s, upon removal of the toxin (Fig. 3 H). Fig. 3I and 3J are the current-voltage curves which indicate that Kv1.1 currents were activated at around −30 mV. From the constructed current/voltage relationship (*I*_test_/*V*_test_), it can be seen that 3 µM HWTX-XI produced a distinct inhibition of Kv1.1 currents at all test pulses; the current-voltage curves indicated that the inhibition of Kv1.1 currents by HWTX-XI was voltage-dependent.

The above results demonstrated that HWTX-XI possesses unique dual biological functions, in that it not only inhibits serine proteinase, but also acts as a blocker of voltage-sensitive K^+^ channels.

### Solution Structure of HWTX-XI

**Figure 4:**
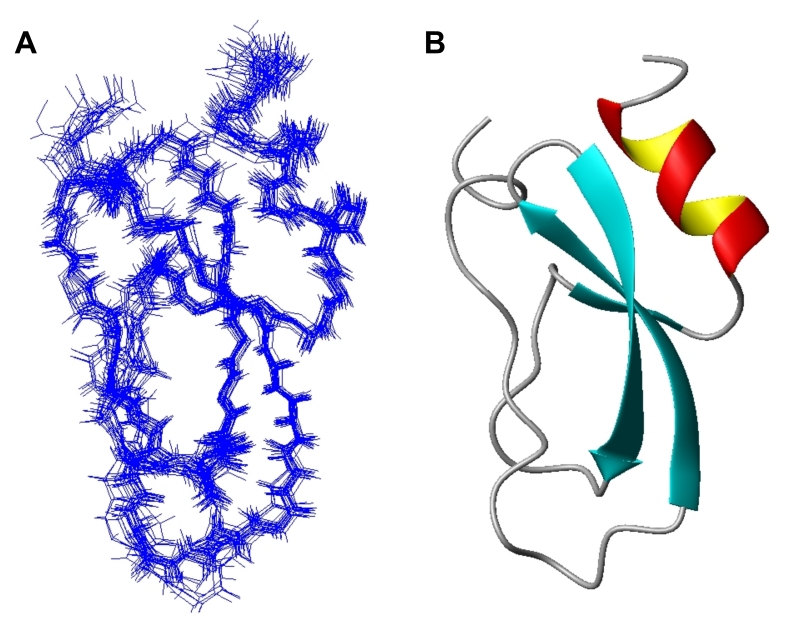
NMR solution structure of HWTX-XI. (A) Superposition of the 20 structures with lowest total energy from final CNS v1.1 calculation. (B) Ribbon presentation of the backbone of HWTX-XI.

Homonuclear ^1^H 2D-NMR and heteronuclear multidimensional NMR experiments were conducted with normal and isotopically labeled HWTX-XI, respectively( Figure. S1, S2 and S3 and Table S1 in Supplemental Materials). The NMR data used for structural calculations are summarized in Table 1. The structure of HWTX-XI was determined by using 1297 intramolecular distance constraints, 44 phi (φ) dihedral constraints, 32 psi (ψ) dihedral constraints, and 29 H-bond restraints. A family of 20 accepted structures with the lowest energies and the best Ramachandran plots was selected to represent the three-dimensional solution structure of HWTX-XI. The structures have no distance violations greater than 0.3 Å and no dihedral violations greater than 3.0°. Analysis of the structures by PROCHECK_NMR showed that more than 80.8% of non-Pro, non-Gly residues lie in the most favored regions, 16.2% in additionally allowed regions, and 3.0% in generally allowed regions of the Ramachandran plot (Fig. S3). A converged representation of the backbone atoms of the 20 best structures of HWTX-XI is shown in Fig. 4A. The root mean square deviations to the mean structure are 1.9±0.21Å and 1.03±0.02 Å for all heavy atoms and for backbone atoms, respectively (Fig. 4B).

**Table 1:** Structural statistics for the family of 20 structures of HWTX-XI.

Experimental constraints	Number
Intra-residue NOE (i−j = 0)	348
Sequential NOE (|i−j| = 1)	360
Medium range NOE (|i−j|≤5)	206
Long range NOE (|i−j|≥5)	374
Disulfide constraints	9
Dihedral angle (ϕ/ω)	76
Hydrogen bonds	29
**r.m.s. deviation from experimental constraints**	
NOE distance (Å) (1297)	0.0052±0.006
Dihedral angle (deg.) (76)	0.2897±0.0562
**r.m.s. deviations from idealized geometry**	
Bonds (Å)	0.0014±0.00012
Angles (deg)	0.3379±0.0083
**Mean energies (kcal mol^−1^)**	
E_bond_	1.77±5.41
E_angle_	2.56±1.32
E_improper_	2.69±0.38
E_NOE_	2.75±0.70
E_cdih_	0.40±0.15
E_total_	52.22±5.41
**Average r.m.s. differences versus mean structure (Å)**	
Backbone atoms (N, Cα, C)	1.03±0.18
Non-hydrogen heavy atoms	1.9±0.21

The solution structure of HWTX-XI (PDB code: 2JOT) resembles a typical Kunitz-type fold (Fig. 4A,B) and is very similar to the solution structures of BPTI and DTX-K[5], [11]. HWTX-XI has an N-terminal 3_10_-helix from Thr3 to Arg5 and a C-terminal α-helix from Gln45 to Cys52, plus a triple-stranded anti-parallel β-sheet of Glu18-Asn23, Thr26-Ile31, and Lys41-Phe42 connected by several reversals. The two helices are connected by a disulfide bond, Cys4-Cys52. The C-terminal α-helix is also connected to the β-sheet by a disulfide bond, Cys27-Cys48. The disulfide bond linkage of HWTX-XI was first guessed at from its Cys residue motif shared with Kunitz-type peptides, and then determined directly from NOE connections of the β protons of the linked cysteine residues.

### Identification of the key residues for the dual functions of HWTX-XI

**Figure 5:**
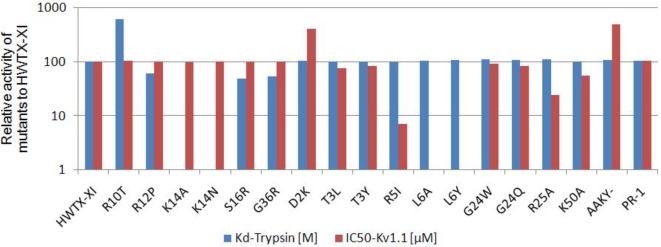
Relative activities of 18 mutants of HWTX-XI. The selection of substituted sites was based on a comparison of the primary structures of HWTX-XI, BPTI, and DTX-K. Values are normalized to the respective activities of wild-type HWTX-XI (100%). The detailed values can be found in supplemental materials Table S1.

To determine the key residues of HWTX-XI for its both functions, 18 mutants were designed according to its solution 3D structure. All the mutant cDNAs of HWTX-XI were constructed through site-directed mutagenesis using HWTX-XI gene as a template and expressed in *S. cerevisiae* strain S-78. The activities of all the purified mutants for both trypsin inhibition and blockage of voltage-sensitive K^+^ channels were measured (Table 2). Fig. 5 is a bar chat illustration of the results according to the data of Table 2. The results show that K14 is mainly responsible for the inhibition function, because the mutation K14N led to a ≈10^5^-fold reduction in inhibitory potency, while K14A showed no binding activity to trypsin at all, as measured by ITC. The surrounding residues, such as R12, S16, F17 and A15, make minor contributions to the binding activity. With regard to to K^+^ channel blocking, the residue Leu 6 seemed to be critical, as its mutation to Ala or Tyr led to a ≈200-fold reduction in inhibitory potency. The mutation R5I reduced this activity ≈14-fold indicating that it has a secondary role in the blocking function. The results shown in Fig. 5 also indicated that none of the mutation in the loop region of HWTX-XI influenced the inhibitory potency toward Kv1.1 channels and that the mutants of the key residues for Kv1.1 channel blockage have no effect on trypsin inhibition activities. These results revealed that there are two separate sites, corresponding to the two types of activities, located on the two ends of the cone-shape molecule HWTX-XI and that they are functionally independent.

**Table 2:** Double functions of HWTX-XI and its mutants.

Protein	to trypsin			to Kv1.1
	*N*	*Kd* [M]	ΔH [kcal/mol]	IC_50_ (mM)
wt	1.07	2.3×10^−10^	−8.94	2.57
aBPTI	1.17	6.57×10^−9^	−6.17	136
bDTX-K	–	–	–	0.01
R10T	0.92	6.58×10^−11^	−12.5	2.49
R12P	1.14	4.15×10^−10^	−7.36	2.61
K14N	0.9	1.95×10^−6^	−2.41	2.66
K14A	–	–	–	2.61
S16R	1.1	2.77×10^−10^	−7.83	2.53
G36R	1.12	9.7×10^−10^	−8.35	2.55
D2K	1.02	2.4×10^−10^	−8.81	0.65
T3L	1.04	2.35×10^−10^	−8.79	3.42
T3Y	1.05	2.2×10^−10^	−8.83	3.15
R5I	1.01	2.3×10^−10^	−8.78	36.5
L6A	1.03	2.3×10^−10^	−8.69	500
L6Y	0.98	2.3×10^−10^	−8.88	481
G24W	1.06	2.2×10^−10^	−8.87	2.81
G24Q	1.02	2.5×10^−10^	−8.92	3.07
R25A	1.01	2.4×10^−10^	−8.76	10.8
K50A	1.05	2.3×10^−10^	−8.94	4.62
cAAKY-	1.05	2.4×10^−10^	−8.99	0.53
dPR-	1.02	2.3×10^−10^	−8.9	2.46

The binding parameters of HWTX-XI and its mutants to trypsin were measured by Isother Titration Calormetry. The experiments were performed in 20 mM HEPES, pH 7.5, at 25°C. Errors in *N* and *ΔH* values are ±10%, and in *Kd* values are ±30%. The IC_50_ values of HWTX-XI and its mutants effect on Kv1.1 channels were carried out in *X. laevis* oocytes. Errors in IC_50_ values are ±10%.

–, no effect detected. ^a^ a potent trypsin inhibitor purified from bovine pancreas [15]; ^b^ a potent blocker for Kv1.1 purified from the venom of *Dendroaspis polylepis* [25]; ^c^ substitute for 1-IDT-3 in HWTX-XI; ^d^ adding two residues ahead of the sequence of HWTX-XI.

**Figure 6:**
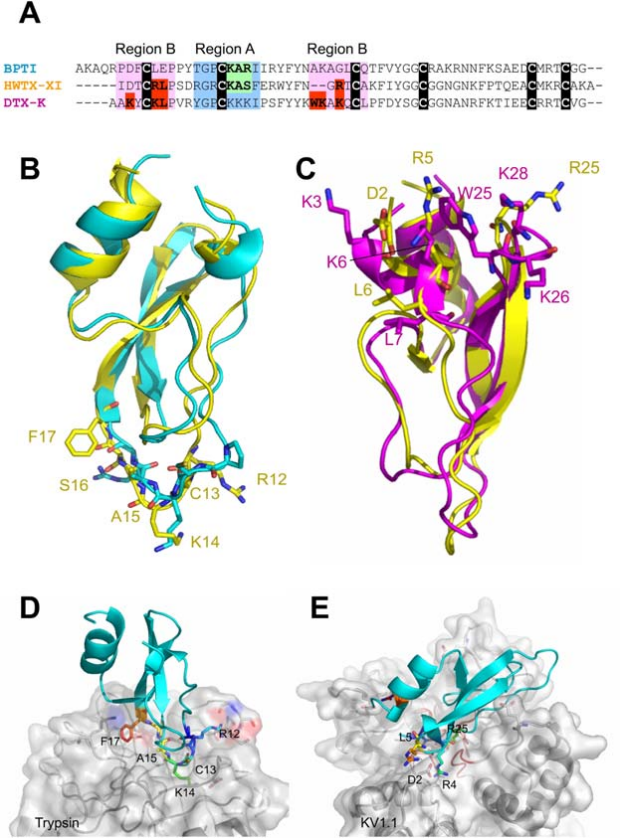
Structural comparison between HWTX-XI with BPTI and DTX-K and docking superimposition of HWTX-XI with Trypsin and pore region of KV1.1. (A). Sequence alignment of PBTI, DTXK and HWTX-XI. (B). Structural superimposition of HWTX-XI in yellow with PBTI in cyan. (C) Structural superimposition of HWTX-XI in yellow with DTX-K in magentas. (D) Docking superimposition of HWTX-XI with trypsin. (E) Docking superimposition of HWTX-XI with pore region of KV1.1. All key residues of HWTX-XI are represented as stick and coloured in rainbow. Some residues of trypsin and Kv1.1 are presented as sticks and the parts of the surface rounding the pore of the channel are colored red.

From the above results we can get some clues to explain why HWTX-XI is stronger than BPTI for the inhibitory activity to trypsin and weaker than DTX-K for the Kv channel blockage. Fig. 6A–C are the sequence and structure comparison between HWTX-XI with BPTI and DTX-K. Fig. 6D and E illustrate the docking superimposition of HWTX-XI with Trypsin and pore region of KV1.1. The comparison indicated possible reasons for why HWTX-XI is a stronger trypsin inhibitor than BPTI. First, the replacement of R21 in BPTI by a smaller residue, S16 reduces steric hindrance and facilitating the approach and interaction of K14 with the negatively charged residue Asp189 at the bottom of the P1 pocket of trypsin. Second, in comparison with the I22 of BPTI, the F17 of HWTX-XI with a larger side chain (phenyl) may provide a broader hydrophobic surface to interact with F41 and K60 of trypsin, which form a hydrophobic pocket. Third, the long side chain of R12 provides a hydrophobic surface and its amino groups fit perfectly the positive pocket formed by the hydroxyls of K145, S146 and G148 in trypsin, and the potential hydrogen bonds between them may strengthen binding to the inhibitor.

The structural superimposition of HWTX-XI and DTX-K (Fig. 6C) and the docking result (Fig. 6E) indicate that, residue Lys3 in DTX-K, which is a key residue for channel binding, is substituted by an Asp residue in HWTX-XI which has a negatively charged and short side chain [18], [19]. This mutation may contribute to the decline of blocking activity. However, the mutation D2K, which was expected to possess potent affinity like DTX-K, increased the activity by only about 4-fold. A possible explanation is that the K residue of the mutant cannot present its side chain to the molecular surface. Another reason for the relatively low blocking activity is that the residues corresponding to W25 and K26 of DTX-K, which may provide a hydrophobic surface for binding to the turret of Kv1.1 subunits are missingin HWTX-XI (Fig. 6 A,C).

### Discovery of a Superfamily of KTTs in Spiders

**Figure 7:**
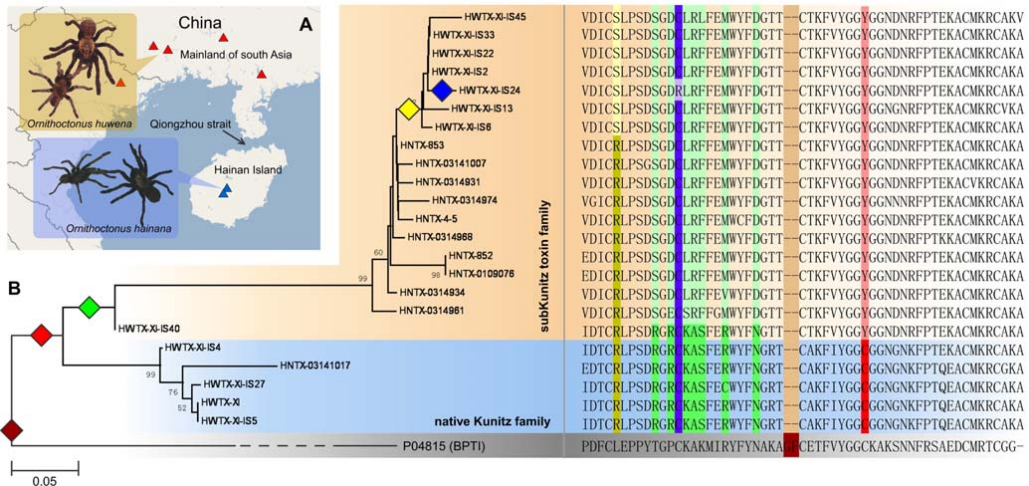
Sequences and evolution of the superfamily of Tarantulas KTTs. (A) The distribution of the *Ornithoctonus huwena* and *Ornithoctonus hainana*. The red and blue triangles indicate the regions in which these spiders are found. (B) A minimum evolution (ME) tree of spider KTTs with sequence alignment. The sub-Kunitz toxin family and native KTT family are marked by orange and light blue backgrounds. The colored diamonds indicate the possible key events of the evolution of spider KTTs: brown, the loss of two amino acids after the recruitment event occurred; red, the substitution of the fourth highly conserved cysteine by a Tyrosine; green, a series of sequence divergences; yellow, a duplication event in Ornithoctonus huwena; blue, the loss of the orphan cysteine residue. The key residues mutated in the key events are marked by backgrounds with the corresponding color.

To investigate whether there are other KTTs similar to HWTX-XI in spider venom, we constructed two cDNA libraries of the venom glands of two Chinese bird spiders *O. huwena* and *O. hainana*. As a result, we have found an additional 45 distinct KTT RNA sequences (34 from *O. huwena* and 11 from *O. hainana*) indicating the presence of a KTT superfamily in spider venom (Fig. 7 and Text S1).

*Ornithoctonus huwena* and *Ornithoctonus hainana* are two primitive spiders distributed on the mainland of south China and Hainan Island respectively (Fig. 7A) [20], [21]. Ecological and molecular evolution evidence supports that they share an ancestor species and derive from the first part of the fourth epoch. In this period, the formation of the Qiong Zhou straits separated them into two groups.

Sequence alignment of novel cDNA sequences of KTTs indicates that they can be categorized into two groups according to the number of disulfide bonds (Fig. 7B and Supplemental materials). The first group follows the native Kuntiz architecture, whereas the second has lost the highly conserved cysteine bridge by a C34Y (TGC->TAC). Members of this group have been designated as sub-Kunitz-type toxins.

Development of a protein family is an extremely complex process which may include a series of key events such as gene duplication and sequence divergence [22]. To try better to understand the evolution of the spider KTT family, we constructed a phylogenetic tree based on the sequence alignment and the topology (Fig. 7B). On careful examination of these data, we identified several possible key events. First, the loss of two residues (A29, K30 in BPTI) must have occurred after the recruitment events because all tarantula spider KTTs and no other Kunitz type proteins share the feature. These data also suggest that the KTTs of tarantulas might share a single origin. The second event is the replacement of the fourth cysteine residue of classical KTTs such as HWTX-XI by a tyrosine, which eliminates the 2–4 disulfide bridge to form the sub-Kunitz toxin. The HWTX-XI-IS40 may be a primitive sub-Kunitz toxin because it shares great sequence similarity with classic KTTs. The loss of disulfide bonds was also seen in two cone snail toxins (conkunitzin-S1, S2), tissue factor pathway inhibitor (TFPI), ixolaris from the salivary gland of the tick and trophoblast Kunitz domain protein-3 (TK-3) from cow[9], [23]. It has been reported that a sub-Kunitz type protein can form a hydrophobic core in region A to compensate for the loss of stability provided by the missing disulfide bridge. As a result, loss of the disulfide may not lead to the destruction of the architecture of the Kunitz type molecules[9]. The following key events may include a series of sequence divergences. The most significant modification may be the mutations (R12D, K14L, A15R and X16F– where the X is other residues) in region A and may involve great functional variations. As Figure 7 shows, both *Ornithoctonus huwena* and *Ornithoctonus hainana* have their sub-Kunitz toxin families. High sequence identity between them suggests gene duplication.That they are not shuffled, strongly supports the assumption that the genes for these toxins were constructed independently by two gene duplication events after the species divergence which occurred less than 1.6 MY ago. The last key event is the deletion of the orphan cysteine residue (the second one) in HWTX-XI-IS24. A possible explanation may be the disappearance of strong purifying pressure on the site after its partner was gone.

### Evolutionary trends in KTTs

It is accepted that many toxins have evolved through recruitment events in which a body protein, cellular ancestor protein of toxin who takes effect in body and do notsecrete out, is recruited into the chemical arsenal[14], [24]. In reality, there are two models used to explain the recruitment events leading to development of new toxins. In the first one, the toxin inherits its function from an ancient body proteins. In the second models, a new function is grafted onto an ancient body protein after the recruitment event to fulfill the requirement of toxin.

**Figure 8:**
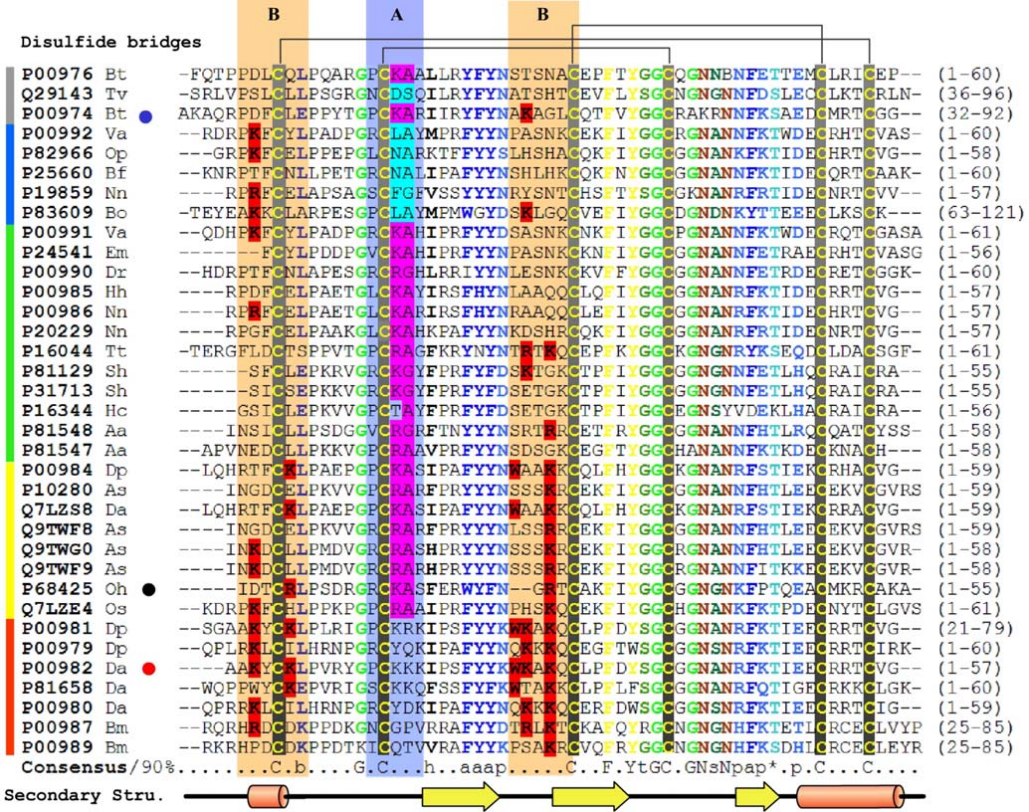
Representative alignment of five classes of Kunitz-type molecules. Different groups classified by function are indicated by colored lines at left: grey, body trypsin inhibitors; blue, chymotrypsin inhibitors in venom; green, trypsin inhibitors in venom; yellow, dual-function toxins; red, K^+^ channel blockers. The alignment was generated by ClustalX (34) and manual editing from the selected protein sequences of the PSI–BLAST (33) search. The 90% consensus sequence was calculated and colored using Chroma (36) Key residues for K^+^ channel blocking and protease inhibition are highlighted in red and cyan, respectively. Regions A and B have light blue and orange backgrounds, respectively. The blue, black and red dots after protein names indicate HWTX-XI, BPTI, and DTX-K, respectively. Capital letters represent amino acids. Lower-case letters represent the following: a, aromatic; b, big; h, hydrophobic; l, aliphatic; p, polar; s, small; t, tiny. The disulfide bridges according to the 3D model are indicated above the alignment. The secondary structure below the alignment is derived from the 3D structure of HWTX-XI (PDB code: 2JOT). Species abbreviations are as follows: Va, *Vipera ammodytes ammodytes*; Sb, *Sarcophaga bullata*; As, *Anemonia sulcata*; Hh, *Hemachatus haemachatus*; Mm, *Macaca mulatta*; Em, *Eristocophis macmahonii*; Os, *Oxyuranus scutellatus scutellatus*; Oh, *Ornithoctonus huwena*; Dp, *Dendroaspis polylepis polylepis*; Da, *Dendroaspis angusticeps*; Nn, *Naja nivea*; Bt, *Bos taurus*; An, *Anoplius samariensis*; Bo, *Boophilus microplus*; Bf, *Bungarus fasciatus*; Cc, *Caretta caretta*; Op, *Ophiophagus hannah*; Dr, *Daboia russellii siamensis*; Tv, *Trichosurus vulpecula*; Hp, *Helix pomatia*; Aa, *Anthopleura aff. xanthogrammica*; Tt, *Tachypleus tridentatus*.

The KTTs seem to have evolved by way of the second model, in that S1-protease inhibitors are transformed into channel blockers in snakes and spiders. A special feature here is that the new active site is different from and independent of the old one. If we thought the development progress of specific K^+^ channel blocker as a chain with four rings from body inhibitors, secreted inhibitor in venoms, double functional toxin and finally to K^+^ channel blockers, the comparing of the two regions among them may provide a new insight into how a new function (ion channel blocking) may be grafted onto an old Kunitz protein scaffold. For doing that, we constructed a sequence alignment of 35 distinct Kunitz type proteins (Fig. 8) which are categorized into five classes; body trypsin inhibitor (BT), chymotrypsin inhibitor in venoms (CTI), trypsin inhibitor in venoms (TI), double functional toxin (DFT) as well as K^+^ channel blocker (KB) of various species based on functional annotations of Swissprot database. The key residues for the two functions are selected out and highlighted manually. These results show clear patterns. C(R/K) s (Where s is a small residue) in region A is shared by all of TIs with the exception of P16344. In the CTIs, the pattern is replaced by C(B)s (where B is a residue with a large side-chain) to fit a broader hydrophobic pocket in the P1 position of chymotrypsin. However, the patterns disappear in the specific K^+^ channel blockers. A clear trend of enrichment of basic residues is found in key sites which have been shown to form an interacting surface with the ion channel, including K3, K6, K26 and K28 in DTX-K [25]. This implies that a dramatic modification occurs in region A in order to discard the inhibition function when a DFT is finally transformed to a KB. In region B, the sequence divergence is much higher than in the others. The trend may be the result of an underlying positive Darwinian selection for the construction of the ion channel blocking function.

### Different evolutionary patterns of KTTs in spiders and snakes

**Figure 9:**
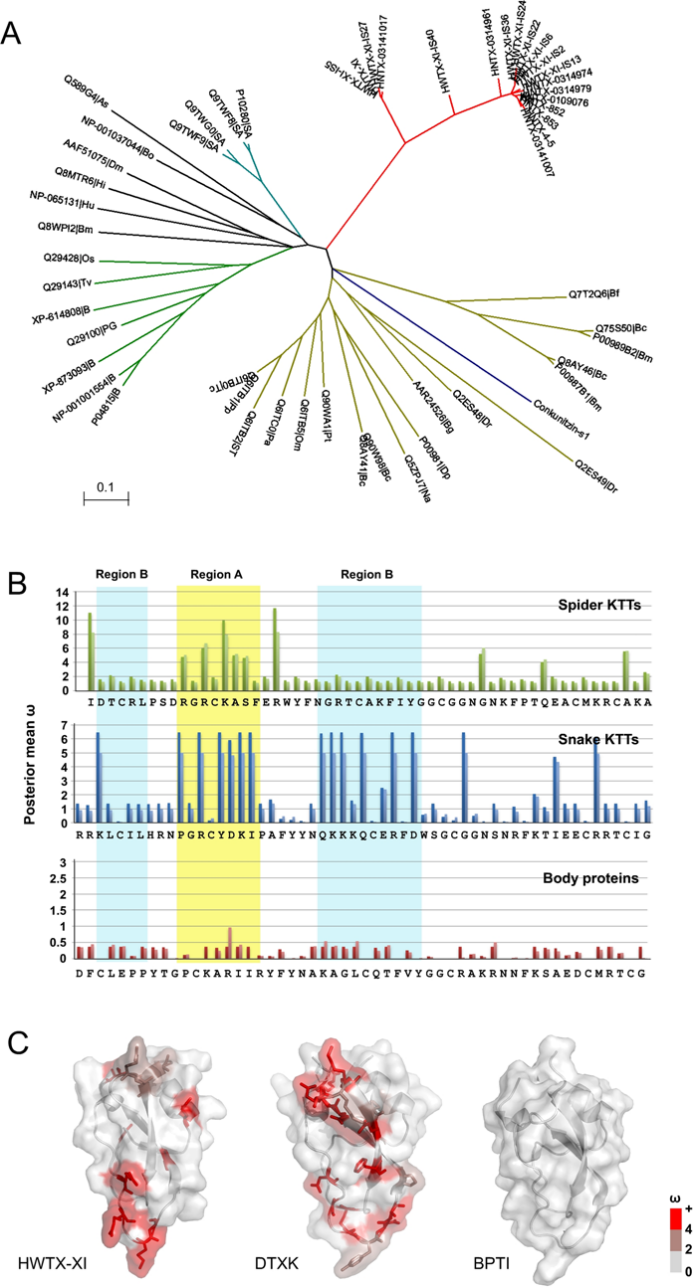
Phylogenetic tree of the KTTs and nonsynonymous/synonymous mutation ratios (ω) comparison. (A), A minimum evolution (ME) tree of mature fragments of KTTs and some Kunitz type body proteins in various taxon groups. The branches are coloured as follows: red, spider KTTs; cyan, sea anemone; green, BPTI homologies from mammals; dark yellow, snake KTTs; blue, conkunitzin from cone snail; black, others. (B), Column charts of the posterior mean nonsynonymous/synonymous mutation ratios (ω) for each site in spider, snake KTTs as well as body Kunitz proteins. In these charts, the deep colour column is based on Model 3 (discrete) and the light one is based on Model 8 (beta&ω>1). Region A and B are marked by yellow and cyan backgrounds, and the secondary structure of HWTX-XI is shown on the bottom. (C), Surface models of representative spider KTTs (HWTX-XI), snake KTTs (DTXK) and Body proteins (BPTI). The residues are colored based on the value of ω in Model 8.

Phylogenetic analyses based on comparison of mature spider KTTs with those inother taxonomic groups, including sea anemone, snake and some body PBTI like proteins [16], [17], [24] has demonstrated that all known Kunitz proteins can be clustered into three clades at the top level and that the spider KTTs are restricted to a clade which appears between the snake and primitive Kunitz type protein clades (Fig. 9A and Text S2). The branch lengths of spider toxins are much longer than that of snake KTTs, suggesting an earlier recruitment event for spider toxins than for snake toxins.

To compare the evolutionary pattern of tarantulas spider KTTs with others, we implemented the “maximum likelihood method” [26] to estimate the nonsynonymous/synonymous mutation ratio (ω) for the three clades ( Table S2, S3 and S4 in Supplemental Materials). The ω ratio is a measure of selective pressure on a protein. An ω ratio significantly higher than 1.0 is convincing evidence for diversifying selection [26]. The presence of a positively selected rate class is detected by likelihood ratio tests (LRTs) for comparing the likelihood of a neutral model with that of a selected model following the suggestions of the PAML manual [27]. Two pairs of models (M1/M2 and M7/M8) were used to test whether the neutral models (M1 and M7) or selection models (M2 and M8) are true. The neutral model constrains ω for each amino acid site to a value between 0 and 1, where ω<0.3 corresponds to purifying selection (selection acting against deleterious mutations) and ω = 1 to neutral evolution, whereas the selected models additional allow positively selected (ω>1) rate classes. For example, the M1 model (neutral) assumes two classes of sites in proteins: the conserved sites (ω = 0) and the neutral sites (ω = 1). The M2 model (selection) adds a third class of sites with ω as a free parameter, thus allowing for sites with x>1. The M7 model (β) allows sites to have 10 different ω ratios in the interval (0, 1), which are calculated from the β distribution with parameters p and q. Model M8 (β and ω) adds an extra class of sites to the β (M7) model and allows the sites to have ω>1. Usually, the model that fit the data better has greater likelihood scores.

The results show that the two selected models (M2 and M8) significantly increase the likelihood scores compared with null models in the case of the KTTs of snake and spider, but not the PBTI like body proteins, suggesting adaptive molecular evolution acting on the KTTs at the sequence level. In contrast, the rather low ω ratio of body proteins (e.g. ω = 0.131 in one ratio model) indicates strong negative selection acting on them (Table S2 in the supplemental materials). The posterior mean ω values of each site of three groups in Model 3 (discrete) and Model 8 (β & ω) are plotted to indicate the sites under positive selection (Fig. 9B and C).As shown, none of the residues in body proteins have an ω ratio higher than 1.0. In the KTTs of snake, residues with a high ω ratio (hω residue) cluster in the region A and region B. In spider KTTs, lower hω residues were found. Most of them cluster in region A and none of them are located in region B, with the exception of I1, the N-terminal residue of HWTX-XI (Supplemental materials Table S2, S3 and S4).

As the Figs 9B and C show, the molecules involved in interactions with the environment usually have a higher evolutionary rate than their body homologs. The distribution of hω residues in KTTs, consistent with alignment results that suggest highly positive Darwinian selection pressure, often focuses on the biologically active region that usually interacts with their ligands. The difference of the distribution of hω residues between tarantula spiders and snakes suggests the distinct functional demands on KTTs and implies a possible reason why snakes, but not spiders, have developed specific K^+^ blockers, though the spider KTTs emerged much earlier. K^+^ channel-blocking activity is important for paralyzing the prey of both snakes and spiders. The absence of positive Darwinian selection pressure in region B of spider KTTs may explain why no specific channel blocking KTTs were found in spider venom.

## Discussion

We report here a new KTT super-family from the venom of spiders and thedetailed study of the structure-function relationship of HWTX-XI, the representative of the spider KTT family. The 45 cDNA sequences of spider KTTs, which doubles the existing dataset of KTTs, and the identification of the functionally and evolutionally independent activity sites of spider KTTs offer us the possibility to explore the KTT evolution.

It is well-known that the main function of animal venom is to kill or immobilize prey. New protein functions are therefore beneficial if they result in more effective toxins. The origin and evolution of animal toxin is a very interesting but still a disputed issue. Now people believe that most toxins have developed from recruitment events of genes from some ‘old’ protein families, according to the phylogenetic analysis of toxin sequences (mainly from that of snakes) and related body proteins. One hypothesis is that a new function is grafted onto ancient body protein after a recruitment event by gene dublication, focal hypermutation and Darwinian selection. For example, the sarafotoxin, a snake toxin with the cardiac arrest activity, is structurally related to endothelins. Sarafotoxin and endothelin-3 possess about 60% amino acid sequence identify. The major differences between them are to be found within the sequences of the inner loop Cys3-Cys11. Scientists believe that sarafotoxin is evolutionally fromthe ancestral endothelin[2], [28]. Another example is α-bungarotoxin, a snake toxin with the activity of blocking neuromuscular transmission. This toxin is structurally related to an ancestral protein, nicotinic acetylcholine receptor binding LYNX. The sequence difference at the N-terminals of the two proteins is the main cause of the neurotoxicity of the α-bungarotoxin [2], [29]. Our work presented here gives another example. The KTTs transform from S1-protease inhibitors to channel blockers in snakes and spiders. Especially, the new active site is different and independent to the old one. The details of the functional evolution remain unknown. Our study indicated that the process of KTT evolution has probably three stages: old functional molecular, bi-functional toxin and new function toxin.

However, a comparison of non-synonymous/synonymous mutation ratios (*ω*) for the two active sites in spider and snake KTTs reveals that the evolution rates of the two active sites are independent. High Darwinian selection pressure acted on the activity sites for both K^+^ channels and serine proteases in snake KTTs but only on the protease site in spider KTTs, suggesting different rates and patterns of KTT evolution. This result indicates that the process of the KTT evolution is very complicated and varying from animal to animal. The reasons for the venomous animals to need protease inhibitors in their venoms and the physiological target of the protease inhibitors still remain unclear to us. It has been proposed that one of the physiological roles of these protease inhibitors is to resist prey proteases to protect their venom protein toxins [14]. It seems that both snakes and spiders got the selective pressure driven by prey proteases. Then we have got a question why snakes have developed strong and specific neurotoxic K^+^ channel blockers while spiders have developed only the dual-functional KTT with weak K^+^ channel blockage activity. We have not got experimental data to answer this question. It is possibly due to the different driving force from the selective pressure. One possible explanation may be that spiders are already equipped with many ICK (inhibitor cystine knot) motif toxins capable of ion channel blocking. For example, based on ATDB database[30] search results, the venom of *O. huwena* contains about 50 different ICK toxins including several potent blockers for Na^+^, Ca^+^^+^ and K^+^ channels[31]. In contrast, few ICK motif toxins have been found in snakes and, except for the Kunitz type K^+^ channel blockers, no other type K^+^ channel blockers have been identified in snakes so far. So it seems that KTTs may comprise the main elements for K^+^ channel blocking for snakes. The weak K^+^ channel blocking activity of spider KTTs should also be developed under Darwinian selective pressure. It is still not sure how the weak K^+^ channel blocking function on the KTTs is beneficial to spiders. We suppose that this weak neurotoxic function may have some synergistic effect with other neurotoxins in the venom, but it need to be proved by experiment data.

The phylogenetic analysis of the KTTs of different taxonomic groups with that of some BPTI like body proteins (Figure 9 A) demonstrated that the development of neurotoxic specific snake KTTs is the later recruitment event than that of spider KTTs. The detailed process of the KTT evolution is still not clear. To better understand the KTT evolution, people need more sequence data of DNA and protein of KTT superfamilies from different venomous animals and more information of the structure-function relationship of KTTs with different functions.

### Concluding Remarks

The present study reports on the dual activity functions and the solution structure of HWTX-XI, a representative Kunitz-type toxin identified for the first time in a tarantula. Structural and functional analyses of 18 mutants revealed two independent binding sites corresponding to the dual activities of HWTX-XI: strong inhibition of serine proteases and weak inhibition of Kv channels. Using HWTX-XI as an example, we discovered a distinct KTT superfamily in two spider species. Comparison of non-synonymous/synonymous mutation ratios (*ω*) for each site in spider and snake KTTs as well as in body Kunitz proteins revealed that the evolution rates of the two active sites are independent. High Darwinian selection pressure acted on the binding sites for both Kv channels and serine proteases in snake KTTs but only on the protease site in spider KTTs. No Darwinian selection pressure was detected for the body proteins, suggesting different rates and patterns of evolution. These results not only introduce a new superfamily of KTTs in spiders, but also provide insights into how new functions can be grafted onto old protein scaffolds and how Darwinian selection pressures affected KTT evolution.

## Materials and Methods

### Collection of Spider Venom

Adult female specimens of *Ornithoctonus huwena* and *Ornithoctonus hainana* were collected in Guanxi and Hainan province of China respectively. The venom was collected by using an electro-pulse stimulator. The two output electrodes of the stimulator were contacted the both sides of the root part of a chelicerae of the spider. Physiological saline was used to enhance electrical contact. Electrostimulation of 36∼80 V, 25∼80 Hz with the pulse time of 0.7 ms was applied across the chelicerae. Expressed venom was collected from the fang tips with a glass vial, and was then freeze-dried[20]. Detail can be found in Methods S1.

### Purification and amino acid sequence analysis of HWTX-XI

HWTX-XI was purified from the venom *of* the spider *O. huwena* using a combination of ion-exchange chromatography and reverse-phase high pressure liquid chromatography (HPLC) as previous described [32]. The molecular mass was determined using a Voyager- DE™ STR MALDI-TOF mass spectrumeter of ABI Company. The entire amino acid sequence was obtained by automated Edman degradation using a 491 pulsed-liquid-phase sequencer from Applied Biosystem Inc.

### Cloning and Sequencing of cDNA encoding HWTX-XI and the mutants

The full-length cDNA of HWTX-XI was amplified from total cDNAs of venomous glands of the spider *O. huwena* Wang using the 3′- and 5′- RACE (rapid amplification of cDNA ends ) as described previously[33]. PCR primers (sense, 5′-TT(T/C)GA(A/G) (A/C)G(A/T/C/G)TG GTA(T/C)TT(T/C)AA(C/T)-3′, 5′-TG(T/C)GC(A/T/C/G)AA(G/A)TT(T/C)AT(A/C/T)TA(T/C)GG-3′, anti-sense, 5′-AATGCTCTGACTGTGTTCCG-3′, 5′-TCTTTTCATGCAGGCCTCTTG-3′),were designed according to the amino acid sequence of HWTX- XI. PCR techniques were applied for the site-directed mutagenesis using HWTX-XI gene as a template. The gene encoding the mature peptide of HWTX-XI and its 18 mutants were cloned into the expression vector pVT102U and transformed into the *S. cerevisiae* strain S-78. The supernatant was collected, purified and identified as mentioning above.

### cDNA library construction

Total RNA was extracted from the venom glands from two spider species. cDNA were prepared from total RNA following the standard protocol. The double-stranded (ds) cDNA molecules were digested by restriction enzyme *Sfi*I and inserted into a pDNR-LIB vector and subsequently electrotransformed into *E. coli* DH10B. Randomly picked clones from each library were sequenced for obtaining the cDNAs encoding KTTs. Then, some oligonucleotide primers were designed according to the conserved 3′ untranslated regions of observed spider KTTs for PCR to screen the cDNAs of KTTs.

### Screening cDNA of spider KTTS

The cDNA from venom gland cDNA libraries of O. huwena Wang and O. hainana was used respectively as template for PCR to screen the cDNAs encoding spider toxin peptides with a Kunitz-type motif. Two oligonucleotide primers, the 5′ PCR Primer (5′-AAGCAGTGGTATCAACGCAGAGT-3′) from a CreatorTM SMART TM cDNA Library Construction Kit in the sense direction, and a specific primer (5′-GTCAAGTAGGTG(C/T)TGACGAT-3′) designed according to the conserved 3′ untranslated regions (UTR) of sequenced peptides with a Kunitz-type motif from venom gland cDNA libraries of O. huwena Wang and O. hainana, in the antisense direction, were used in PCR reactions.

The PCR conditions were: 94°C for 5 min, followed by 30 cycles of 94°C for 1 min, 53°C for 1 min and 72°C for 1 min, concluding with a final step of 72°C for 10 min. The resulting PCR fragments were cloned into pGEM®-T Easy vector (Promega), and at least 120 clones from each reaction were sequenced (Methods S2 and Text S1).

### Assay of serine protease inhibitory potency

Trypsin assay was performed in 100 mM Tris-HCl (pH 8.0), containing 20 mM CaCl_2_ and 0.05% triton X-100. Trypsin was incubated with various amount of HWTX-XI for 10 min at a final concentration of 0.4 µM. The substrate Benzoyl-L-arginine-p-nitroanilide (BAPNA) was used at a final conaentration of 0.4 mM. The remain serine protease activity was monitored at 405 nm. The association and dissociation of HWTX-XI with trypsin and α-chymotrypsin was measured with a BIAcore X instrument (BIAcore AB). HWTX-XI was coupled to a carboxymethylated dextran CM5 sensor chip. The binding assay was performed with a constant flow rate of 20 µl/min at 25°C through the chip. The binding kinetics was fitted with BIAevaluation version 3.1. In titration calorimetry experiments, HWTX-XI or its mutants (0.001 mM, dissolved in 20 mM HEPES, pH 7.5) in the calorimetric cell was titrated with 0.01 mM trypsin buffer solution at 25°C. The raw calorimetry data were collected and analyzed by Origin Version 7.0 software.

### Patch clamp recording on rat dorsal root ganglion neurons

Rat DRG neurons were acutely dissociated and maintained in a short-term primary culture according to the procedures adapted from Xiao *et al*.[32]. Briefly, the rat dorsal root ganglia were removed quickly from the spinal cord, and then they were transferred into Dulbecco's modified Eagle's medium containing trypsin (0.3 g/liter, type III), collagenase (0.7 g/liter, type IA) to incubate at 34°C for 22 min. Trypsin inhibitor (1.5 g/liter, type II-S) was used to terminate enzyme treatment. The DRG cells were transferred into 35-mm culture dishes (Corning, Sigma) containing 95% Dulbecco's modified Eagle's medium, 5% newborn calf serum, hypoxanthine aminopterin thymidine supplement, and penicillin-streptomycin and then incubated in the CO2 incubator (5% CO2, 95% air, 37°C) for 1–4 h before the patch clamp experiment.Potassium currents were recorded from experimental cells using whole-cell patch clamp technique. The internal solution contained the following (in mM): 120 KF, 20 NMG, 10 HEPES, 10 EGTA, 2 MgATP, 0.5 Li_2_GTP, at pH 7.2. The external bathing solution contained the following (in mM): 130 Choline chloride; 5 KOH; 12 D-glucose; 2 MgCl_2_; 2 CaCl_2_; 10 HEPES; at pH 7.2. After establishing the whole-cell recording configuration, the resting potential was held at-80 mV for at least 4 min to allow adequate equilibration between the micropipette solution and the cell interior. Ionic currents were recorded on EPC-9/10 patch clamp amplifier (HEKA, Lambrecht, Germany). The P/4 protocol was used to subtract linear capacitive and leakage currents. Sodium currents were recorded according to Xiao *et al* [32]. Calcium currents were recorded according to *Wang et al* [34]. All experiments were conducted in accordance with the guidelines established by the Committee on the Use and Care of Animals at the Hunan Province, P. R. China.

### Oocyte manipulation and electrophysiological recording

Capped cRNAs encoding ion channels were synthesized after linearizing the plasmids and performing the transcription by a standard protocal [35]. For in vitro transcription, the plasmids pCI containing the genes for Kv1.1 and Kv2.1 were first linearized with Not I respectively; the plasmid PcDNA3 containing the gene for Kv1.2 was linearized with *Sph* I; the plasmid pCI-neo containing the gene for Kv1.3 was linearized with *Not*I; the plasmid PcDNA3.1containing the gene for Kv4.2 was linearized with *Sma* I; the plasmids pSP64 containing the genes for Kv1.4 and Kv3.1 were linearized with EcoR I; the plasmid pOX containing the gene for mSlo was linearized with Nsb I. Using the linearlized plasmids as templates, cRNAs were synthesized *in vitro* using the large-scale T7, SP6 or T3 mMESSAGE mMACHINE transcription kit (Ambion, USA). Stage V-VI *Xenopus laevis* oocytes were collected from mature female *Xenopus laevis* under anaesthesia by putting on ice. Then the oocytes were defolliculated by treatment with 1 mg/ml collagenase in calcium-free ND96 solution (pH 7.5) containing concentrations of (in mM) 96 NaCl, 2 KCl, 1 MgCl_2_, and 10 HEPES, Between 2 and 24 h after defolliculation, oocytes were injected with 41 nl of 100–500 ng/µl cRNA using a Microprocessor controlled nanoliter injector (WPI, USA). The oocytes were then incubated in OR_2_ solution (pH = 7.5) at 18°C for 1–4 days. OR_2_ solution contains (in mM) 82.5 NaCl, 2.5 KCl, 1 CaCl_2_, 1 Na_2_HPO_4_, 1 MgCl_2_, 5 HEPES, supplemented with 50 mg/L gentamycin sulphate (only for incubation). Whole-cell currents from oocytes were recorded using the two-microelectrode voltage-clamp (TURBO TEC-03X, NPI Electronic, Germany). Voltage and current electrodes (0.4–2 M) were filled with 3 M KCl. The bath solution was ND96 (pH 7.5). Current records were sampled at 0.5 ms intervals after low pass filtering at 1 kHz. Linear components of capacity and leak currents were not subtracted. All experiments were performed at room temperature (19–23°C).

### NMR structure determination of HWTX-XI

The homonuclear 1H two-dimensional (2D) NMR spectra DQF-COSY, TOCSY and NOESY were recorded on a Bruker Avance 800 MHz spectrometer with the unlabeled HWTX-XI sample. The multi-dimensional spectra, including ^15^N-^1^H HNHB, ^15^N-^1^H TOCSY-HSQC and ^15^N-^1^H NOESY-HSQC were recorded on a Bruker Avance 600 MHz spectrometer with the ^15^N labeled sample. All NMR experiments were carried out at 303° K. The spectra were processed with NMRpipe[36] and analyzed by NMRview [37]. The CNS v1.1 program[38] was used to calculate and refine the structures. The statistics on experimental constrains,coordinate precision and stereochemical quality of 20 structures with the lowest energy were analyzed with MOLMOL [39] and PROCHECK[40] (Methods S3).

### Sequence comparison and modeling

The structural coordinates of DTX-K (PDB: 1DTK), BPTI (PDB: 1PIT) and typsin (PDB: 2PTN) were extracted from the PDB database. The KV1.1 pore region was modeled according to the crystal structure of KV1.2 (PDB: 1ZWI) using modeler [41]. The trypsin/toxin complex model is based mainly on the structural alignment to the known coordinates of the BPTI/toxin complex (PDB: 1TAW). Based on key residues information, the model of HWTX-XI was docking to fit the surface of the pocket region of KV1.1 manually for KV1.1/toxin complex construction (Methods S4).

### Evolutional analysis

133 non-redundant body Kunitz-type proteins and KTTs of other species were selected from SwissProt and GenBank database by PSI-BLAST[42]. Spider KTTs are from cDNA library sequencing. Sequences without signal peptide or with very high sequence similarity to others were omitted and finally 38 distinct proteins and 32 cDNA sequences were selected out for further analysis.

Sequence alignment was created by ClustalX 1.83 [43]. The phylogenetical trees were implemented in MEGA3.1 [44] and evaluated by 1000 interior branch test. The ω ratio was estimated by the CODEML program of the PAML package [27]. After ML estimates of parameters were obtained, the empirical Bayesian approach was used to calculate the posterior mean ω ratio and the probability belongs to a given rate class for each site. The detailed results are summarized in supplemental materials Methods S5 and Table S2, S3 and S4.

## Supporting Information

Methods S1 Spider and venom collection
(0.03 MB DOC)

Methods S2 Clone and Mutations
(0.03 MB DOC)

Methods S3 Structure calculation
(0.02 MB DOC)

Methods S4 Sequences collection and modeling
(0.05 MB DOC)

Methods S5 Evolution
(0.04 MB DOC)

Figure S1 Sequential assignment of HWTX-XI by 3D 15N-1H NOESY-HSQC. Nearly complete dαN and dNN sequential connectivity was established throughout the sequence of HWTX-XI except for I1, P7, G34 and P43.
(2.87 MB TIF)

Figure S2 2D 15N-1H HSQC spectrum of HWTX-XI. Amide NH assignments are annotated with the one-letter amino acid code and the sequence number, assignments of side-chain NH and NH2 groups are also shown. HN positions are shown by the dashed lines.
(0.72 MB TIF)

Figure S3 Analysis of the family of 20 structures using the program PROCHECK
(3.60 MB TIF)

Table S1 Chemical shifts of the assigned protons of HWTX-XI.
(0.11 MB DOC)

Table S2 Parameter estimates and likelihood ratio statistics (2△l) for the BPTI like body proteins
(0.03 MB DOC)

Table S3 Parameter estimates and likelihood ratio statistics (2△l) for the snake KTTs
(0.04 MB DOC)

Table S4 Parameter estimates and likelihood ratio statistics (2△l) for the spider KTTs
(0.03 MB DOC)

Text S1 The txt file contains 48 cDNA sequences of Spider KTTs with FASTA format and can be downloaded as separated file named as spider_KTTs.fas.total_cDNA.fas.
(0.02 MB TXT)

Text S2 The txt file contains aligned 56 mature peptide sequence, which were used to construct phylogenetic tree in Figure 3a of main text with FASTA format. It can be downloaded as separated file named as aligned_mature.fas.
(0.00 MB TXT)
