# Correction: Discovery of a Distinct Superfamily of Kunitz-Type Toxin (KTT) from Tarantulas

**DOI:** 10.1371/annotation/a7561dde-3b04-4fd9-9267-463b23cc7dd0

**Published:** 2008-11-03

**Authors:** Chun-Hua Yuan, Quan-Yuan He, Kuan Peng, Jian-Bo Diao, Li-Ping Jiang, Xing Tang, Song-Ping Liang

The sequence shown in Figure 1C is incorrect. Please view the entire corrected figure here:

**Figure 1:**
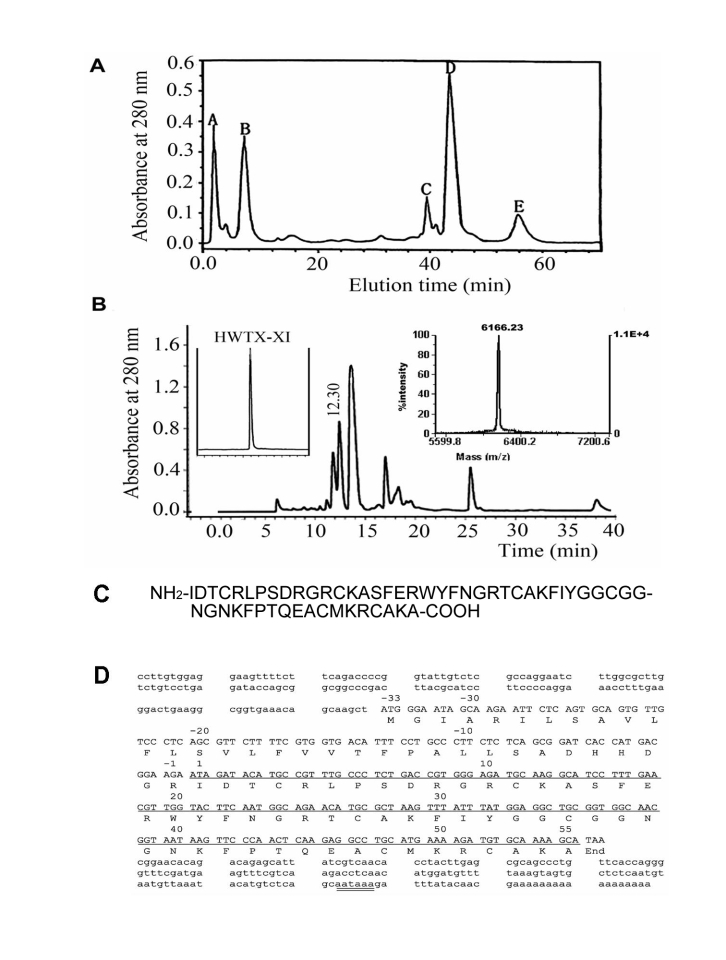
Purification and amino acid sequence of HWTX-XI. (A) Ion-exchange HPLC chromatograph of crude Ornithoctonus huwena venom. The column (Waters Protein-Pak CM, 10 mm×100 mm) was equilibrated with 0.1 M sodium phosphate buffer, pH 6.8, the crude venom (10 mg in 2 ml) was loaded and eluted at a flow rate of 3 mL/min, using a gradient of 0–81% buffer B (1 M NaCl, 0.1 M sodium phosphate buffer, pH 6.8 ) over 60 min. Then the peak E was applied to Vydac C18 reverse phase column (4.6 mm×250 mm) equilibrated with 0.1% trifluoroacetic acid (B), using a gradient of 20–40% buffer C (0.1% trifluoroacetic acid in acetonitrile) over 45 min. The fraction of retention time 12.30 min containing HWTX-XI was further purified by Vydac C18 rpHPLC as shown in the left inset. The molecular mass of HWTX-XI was determined by MALDI-TOF mass spectrometry (right inset of B). (C) Amino acid sequence of HWTX-XI determined by Edman degradation. (D) The oligonucleotide sequence of HWTX-XI cDNA. The cDNA encoding the mature peptide are underlined. The polyadenylations signal, AATAAA, is double underlined.

