# Correction: Discovery of a Distinct Superfamily of Kunitz-Type Toxin (KTT) from Tarantulas

**DOI:** 10.1371/annotation/db7652dc-4328-48ea-9244-67e831ae0e0e

**Published:** 2008-11-04

**Authors:** Chun-Hua Yuan, Quan-Yuan He, Kuan Peng, Jian-Bo Diao, Li-Ping Jiang, Xing Tang, Song-Ping Liang

There were a number of errors in the legend for Figure 3, panels D-H. The corrected text is shown here:

**Figure 3:**
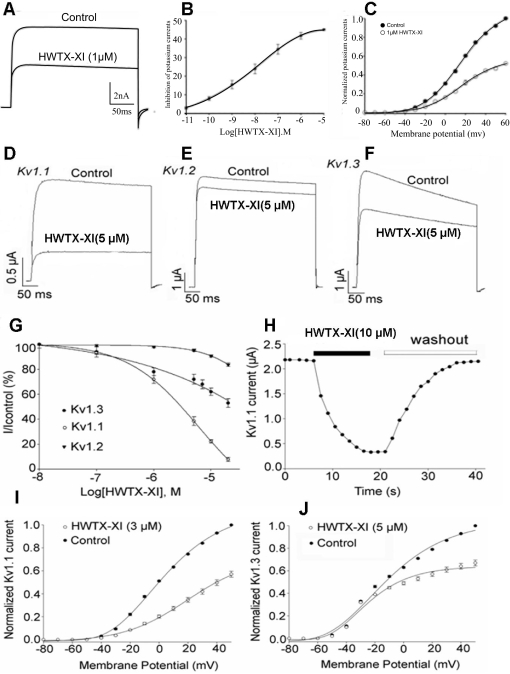
Effects of HWTX-XI on Kv channels. (A) 1 µM HWTX-XI evidently reduced the control potassium currents amplitude in rat DRG neurons by 41.7±1.8% (n = 5). (B) Concentration-response relationship for HWTX-XI inhibition of potassium currents expressed on rat DRG neurons. Each data point (mean±S.E.) arises from 5–7 cells. The solid line through the data is a fit of *I*/*I_max_* = 1/[1+exp(*C*–*IC_50_*)/*Κ*]. (C) Effect of HWTX-XI on steady-state current-voltage relationship of potassium channels on rat DRG neurons. DRG cells were held at −80 mV and stepped to test potentials of −80 to +60 mV (mean±SD, *n* = 4). (D–F) 5 µM HWTX-XI evidently reduced the control potassium currents (Kv1.1, D; Kv1.2, E; Kv1.3, F) amplitude in *X. laevis* oocytes. (G) Concentration-response relationship for HWTX-XI inhibition of potassium currents expressed in *X. laevis* oocytes. Each data point (mean±S.E.) arises from 5–7 cells. The solid line through the data is a fit of *I*/*I_max_* = 1/[1+exp(*C*−*IC_50_*)/*Κ*]. (H) At a concentration of 10 µM, HWTX-XI produced a rapid (τ<12±2 s for steady-state inhibition, n = 5) inhibition which is readily reversible with the time constant of 21±3 s upon removal of the toxin. (I–J) The current-voltage curves of Kv1.1 (I) and Kv1.3(J) currents activated by 3 µM and 5 µM HWTX-XI respectively.

