# Correction: Discovery of a Distinct Superfamily of Kunitz-Type Toxin (KTT) from Tarantulas

**DOI:** 10.1371/annotation/5a7fb62a-94b0-4a9e-9031-b391502df41a

**Published:** 2008-11-04

**Authors:** Chun-Hua Yuan, Quan-Yuan He, Kuan Peng, Jian-Bo Diao, Li-Ping Jiang, Xing Tang, Song-Ping Liang

In Figure 2D and 2E, the lower half of the vertical axes are mislabeled. There was also factual error in the legend for Figure 2D-E. Please view the entire corrected figure and legend here:

**Figure 2:**
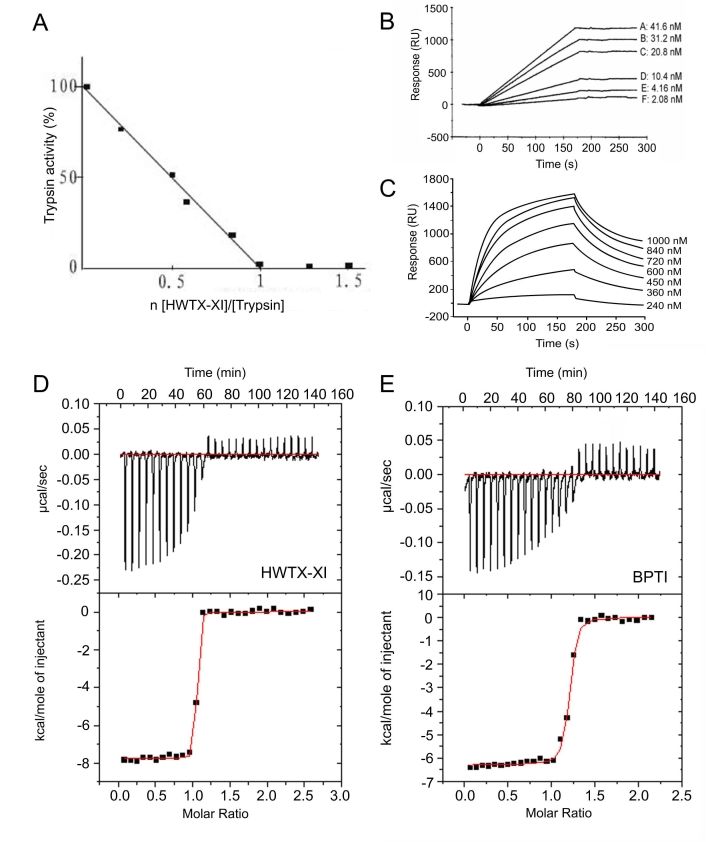
The inhibitory activities of HWTX-XI to proteinases. (A) The inhibition stoichiometry of HWTX-XI to trypsin. Trypsin assay was performed in 100 mM Tris-HCl (pH 8.0), containing 20 mM CaCl_2_ and 0.05% triton X-100. Trypsin was incubated with various amount of HWTX-XI for 10 min with the substrate Benzoyl-L-arginine-p-nitroanilide (BAPNA) at a final conaentration of 0.4 mM. The protease activity was monitored at 405 nm. (B) BIAcore analysis of immobilized HWTX-XI binding to trypsin (2.08–41.6 nM) with a BIAcore X instrument (BIAcore AB, Uppsala, Sweden). HWTX-XI (11.5 µM,desolved in 10 mM sodium acetate,pH 5.5)was coupled to a carboxymethylated dextran CM5 sensor chip. The binding assay was performed with a constant flow rate of 20 µl/min at 25°C. (C) BIAcore analysis of immobilized HWTX-XI binding to α-chymotrypsin (240 nM to 1000 nM). (D) and (E) Isothermal titration calorimetry data of HWTX-XI (D) and BPTI (E) titrated with trypsin. Experiments were conducted with a VP-ITC system at 25°C with stirring at 300 rpm. Top, raw data (cal/s vs. time) showing heat release upon injection of 0.1 mM trypsin into a 1.4-ml cell containing 0.01 mM HWTX-XI (D) or BPTI (E). Bottom, integration of the raw data yields the kcal/mol vs. molar ratio.

