# Supplementary material for: Discovery of a Distinct Superfamily of Kunitz-Type Toxin (KTT) from Tarantulas
Source: PLoS One. 2008 Oct 15;3(10):e3414. doi: 10.1371/journal.pone.0003414 (PMC2561067; doi:10.1371/journal.pone.0003414)
Supplement: Methods S1 — Spider and venom collection (0.03 MB DOC) [file pone.0003414.s001.doc]

**Methods S1**:

- Spider and venom collection

In the summer of 1991, Prof. Wang identified a large hairy spider of light brown body color in hilly area of Ninming country of Guanxi province as a new species of the family Theraphosidase and named it Selenocosmia huwena(1). Recently the taxonomic classification of this spider was reevaluated, the spider was proposed to be transferred to the genus Ornithoctonus and was renamed Ornithoctonus huwena (2). They widely distribute in forests of the mainland of south Asia. Comparing with Selenocosmia huwena, they are litter small and have a deep body color. Ecological and molecular evolution evidences support that they share an ancestor species and derivate on the forepart of the fourth epoch (160MYs ago). In this period, the QiongZhou strait was constructed and separated them into two groups (2). However, the components of venom are very different between them.

The methods of venom collection are similar between them. The venom was collected by using an electro-pulse stimulator. The two output electrodes of the stimulator were contacted the both sides of the root part of a chelicerae of the spider. Physiological saline was used to enhance electrical contact. Electrostimulation of 36~80 V, 25~80 Hz with the pulse time of 0.7ms was applied across the chelicerae. Expressed venom was collected from the fang tips with a glass vial, and was then freeze-dried(3).

**References**

*1. Wang, J. F., Peng, X.J, Xie, L. P. (1993) Acta Sci. Nat. Univ. Norm. Hunan.* ***16****, 4*

*2. Zhu, M. S., Song, D.X. (2000) J. Hebei University* ***20****, 3*

*3. Liang, S. (2004) Toxicon* ***43****(5), 575-585*
