# Supplementary material for: Discovery of a Distinct Superfamily of Kunitz-Type Toxin (KTT) from Tarantulas
Source: PLoS One. 2008 Oct 15;3(10):e3414. doi: 10.1371/journal.pone.0003414 (PMC2561067; doi:10.1371/journal.pone.0003414)
Supplement: Methods S2 — Clone and Mutations (0.03 MB DOC) [file pone.0003414.s002.doc]

**Methods S2**

- Cloning and Sequencing of cDNA encoding HWTX-XI

The full-length cDNA of HWTX-Ⅺ was amplified from total cDNAs of venomous glands of the spider *O. huwena* Wang using the 3’- and 5’- RACE (rapid amplification of cDNA ends ) as described previously(5). PCR primers (sense, 5’-TT(T/C)GA(A/G) (A/C)G (A/T/C/G)TGGTA(T/C)TT(T/C)AA(C/T)-3’, 5’-TG(T/C)GC(A/T/C/G)AA(G/A)TT(T/C)AT (A/C/T)TA(T/C)GG-3’, anti-sense, 5’-AATGCTCTGACTGTGTTCCG-3’, 5’-TCTTTTCAT GCAGGCC TCTTG-3’ ), were designed according to the amino acid sequence of HWTX-Ⅺ. The PCR products were purified and cloned into the pGEM-T easy vector for sequencing. DNA sequencing was performed by Bioasia Inc.

- Construction and expression of HWTX-XI and its mutants

The gene encoding mature peptide of HWTX-XI was cloned through the flanking *Xba*I and *Hind* III restriction sites into the expression vector, pVT102U. PCR techniques were applied for the site-directed mutagenesis using HWTX-XI gene as a template. The reconstructed gene was cleaved with restriction endonucleases *Xba*I and *Hin*dIII, ligated to vector pVT102U, and transformed into the *S.* *cerevisiae* strain S-78. The transformant was grown at 30℃ in YPD medium for another 3 to 4 days. The supernatant was collect and purified using a combination of cation ion-exchange chromatography and reverse-phase high pressure liquid chromatography (HPLC). Their molecular masses were determined by MALDI-TOF mass spectrometer of ABI Company. Detailed results can be found in Table S1.

- RNA extraction and cDNA library construction

Total RNA was extracted using TRIzol (Invitrogen) from the venom glands from each of the Chinese bird spiders *O. huwena* Wang and *O. hainana*. cDNA were prepared from total RNA by using a Creator^TM^ SMART ^TM^ cDNA Library Construction Kit (Clontech) following the standard protocol of the manufacturer.

The double-stranded (ds) cDNA molecules were digested by restriction enzyme *Sfi*I and inserted directionally into a pDNR-LIB vector (Clontech) at the *Sfi*I position and subsequently electrotransformed into *E. coli* DH10B using MicroPulser electroporation apparatus (Bio-Rad). The transformation for the *O. huwena* Wang and *O. hainana* ligations yielded libraries comprising approximately 1.0×10^6^ primary clones each. Randomly picked clones from each library plating were sequenced by the dye terminator method on ABI 377 automated sequencers and sequence analyses were analyzed using MEGA 3.0(6).

**Reference:**

*6. Kumar, S., Tamura, K., and Nei, M. (2004) Briefings in bioinformatics* ***5****(2), 150-163*
