# Supplementary material for: Discovery of a Distinct Superfamily of Kunitz-Type Toxin (KTT) from Tarantulas
Source: PLoS One. 2008 Oct 15;3(10):e3414. doi: 10.1371/journal.pone.0003414 (PMC2561067; doi:10.1371/journal.pone.0003414)
Supplement: Methods S3 — Structure calculation (0.02 MB DOC) [file pone.0003414.s003.doc]

**Methods S3**

- Structure calculation and description

The structure of HWTX-XI was determined by using 1297 intramolecular distance constraints, 44 phi dihedral constraints and 32 psi dihedral constraints (ψ), and 29 H-bond restraints. A family of 20 accepted structures with the lowest energies and the best Ramachandran plots was selected to represent the three-dimensional solution structure of HWTX-XI. The structures have no distance violations greater than 0.3 Å and no dihedral violations greater than 3.0°. Analysis of the structures by PROCHECK_NMR shows that more than 80.8% of non-Pro, non-Gly residues lies in the most favored regions, 16.2% in additionally allowed regions, and 3.0% in general allowed regions of the Ramachandran plot. A representation of the backbone atoms of the 20 best converged structures of HWTX-XI is shown in Fig.2 in main text. For all heavy atoms the root mean square deviation to the mean structure is 1.9±0.21Å, and for the backbone atoms it is 1.03±0.018 Å.

The solution structure of HWTX-XI (PDB code: 2JOT) resembles a typical kunitz-type fold. It composes of an N-terminal 310-helix from Thr3 to Arg5 and a C-terminal α-helix from Gln45 to Cys52, plus a triple-stranded anti-parallel β-sheet of Glu18-Asn23, Thr26-Ile31 and Lys41-Phe42 connected by several reversals. The two helices are connected by a disulfide bond Cys4-Cys52. The C-terminal α-helix is also connected by a disulfide bond Cys27-Cys48.The disulfide bond linkage of HWTX-XI was firstly speculated from its identical Cys residues motif with those of kunitz-type peptides and then determined by direct NOE connections of the β protons of the linked Cysteine residues.
