# Supplementary material for: Discovery of a Distinct Superfamily of Kunitz-Type Toxin (KTT) from Tarantulas
Source: PLoS One. 2008 Oct 15;3(10):e3414. doi: 10.1371/journal.pone.0003414 (PMC2561067; doi:10.1371/journal.pone.0003414)
Supplement: Methods S4 — Sequences collection and modeling (0.05 MB DOC) [file pone.0003414.s004.doc]

**Methods S4**

- Sequences gathering and selection

Using PSI-BLAST(7) with an inclusion threshold of 0.05, we searched the SwissProt databases version 51.4 against the HWTX-XI protein. The search converged after four iterations and retrieved 133 non-redundant protein sequences in total. Sequences with muti-Kunitz domains or highly sequences similarity are omitted. Then 32 distinct KTTs were selected out manually for multiple sequence alignment. Based on annotations in the database, functional these proteins can be categorized into four classes: chymotrypsin inhibitor, trypsin inhibitor, double functional toxin as well as K channel blocker. The alignment was created by ClustalX 1.83(8) with manual adjustment and colored by Chroma(9) (Figure S2).

Raw cDNA sequences of BPTI like body proteins and snake KTTs are obtained by search against NCBI GenBank database using as mature peptides of BPTI, DTXK(10,11) and relative sequences as probe. The significant hits are sifted further, ones which have muti-Kunitz domain or without signal peptide are omitted. Sequences with very low sequence similarity in signal peptide with probes (low than 40%) are filtered out. Additionally, for snake KTTs, peptides from other taxon group are deleted. Spider cDNA library sequencing presents the raw data of spider KTT. For them, sequences with very highly similarity are omitted manually. Finally, 15 BPTI like proteins, 18 snake KTTs and 23 spider KTTs are selected out for statistical analysis.

- Modeling

The structural coordinates of DTX-K (PDB: 1DTK), BPTI (PDB: 1PIT) and typsin (PDB: 2PTN) were extracted from the PDB database. The model of KV1.1 pore region was modeling according to the crystal structure of KV1.2 (PDB: 1ZWI) from the PDB by using modeler 3. Docking model of typsin/toxin complex is mainly based on the structural alignment to known coordinate of BPTI/toxin complex (PDB: 1TAW). On the base of integrating related structure information(12,13), we constructed the KV1.1/toxin complex by docking the model of HWTX-XI to fit the surface of pocked region of the KV1.1 manually. All of structural comparisons and picture drawing ware performed by Pymol (<http://pymol.sourceforge.net/>)(14).

**References**

*7. Altschul, S. F., and Koonin, E. V. (1998) Trends in biochemical sciences* ***23****(11), 444-447*

*8. Thompson, J. D., Gibson, T. J., Plewniak, F., Jeanmougin, F., and Higgins, D. G. (1997) Nucleic acids research* ***25****(24), 4876-4882*

*9. Goodstadt, L., and Ponting, C. P. (2001) Bioinformatics (Oxford, England)* ***17****(9), 845-846*

*10. Wang, F. C., Bell, N., Reid, P., Smith, L. A., McIntosh, P., Robertson, B., and Dolly, J. O. (1999) European journal of biochemistry / FEBS* ***263****(1), 222-229*

*11. Gasparini, S., Danse, J. M., Lecoq, A., Pinkasfeld, S., Zinn-Justin, S., Young, L. C., de Medeiros, C. C., Rowan, E. G., Harvey, A. L., and Menez, A. (1998) The Journal of biological chemistry* ***273****(39), 25393-25403*

*12. Szabo, E., Bocskei, Z., Naray-Szabo, G., and Graf, L. (1999) European journal of biochemistry / FEBS* ***263****(1), 20-26*

*13. Blanc, E., Romi-Lebrun, R., Bornet, O., Nakajima, T., and Darbon, H. (1998) Biochemistry* ***37****(36), 12412-12418*

*14. Wu, Y., Cao, Z., Yi, H., Jiang, D., Mao, X., Liu, H., and Li, W. (2004) Biophysical journal* ***87****(1), 105-112*
