# Supplementary material for: Discovery of a Distinct Superfamily of Kunitz-Type Toxin (KTT) from Tarantulas
Source: PLoS One. 2008 Oct 15;3(10):e3414. doi: 10.1371/journal.pone.0003414 (PMC2561067; doi:10.1371/journal.pone.0003414)
Supplement: Methods S5 — Evolution (0.04 MB DOC) [file pone.0003414.s005.doc]

**Methods S5**

- Toxin phylogenetic analysis

In order to minimize confusion, all proteins sequences are referred to by their Swiss-Prot or NCBI nr database accession numbers (<http://www.expasy.org/cgi-bin/sprot-search-ful>). Comparative sequences were obtained through BLAST searching using representative toxin sequences (http://www.expasy.org/tools/blast/). A total of 36 representative distinct sequences from all 133 KTTs and 7 intracellular Kunitz type sequences were selected out combining with 21 representative spider KTT sequences to construct the phylogenetical tree. Resultant sequence sets were aligned using the program CLUSTAL-X 1.83, followed by visual inspection for errors. Then, the aligned sequences were used to make phylogenetical trees using several different methods (Minimum evolution (ME) and Neighbor Join (NJ)) implemented in MEGA3.1(6), with the pairwise deletion option. The tree was evaluated by 1000 interior branch test resamplings and drawn by MEGA3.1.

- Statistical analysis of sequence divergence

To test for positive selection at single sites of aligned spider, snake KTTs and BPTI like body protein sequences, we performed a maximum likelihood analysis to estimate the nonsynonymous to synonymous rate ratio (ω= K_a_/K_s_) using the CODEML program of the PAML software package (<http://abacus.gene.ucl/software/paml.html>)(15). The presence of a positively selected rate class is detected by likelihood ratio tests (LRTs) for comparing the likelihood of a neutral model with that of a selection model following the suggestions of PAML manual(15). The neutral model with that constrain ω for each amino acid site between 0 and 1, where ω<0.3 corresponds to purifying selection (selection acting against deterious mutations) and ω=1 to neutral evolution, whereas the selection models additional allow positively selected (ω>1) rate classes.

Six different models for ω ratio distribution among sites are used. The M0 model (one-ratio) assures that all sites have the same ω ratio, Model M3 (discrete) use a general discrete distribution with three sites classes, with the proportions p0, p1 and p2 with ω ratios ω0, ω1 and ω2 respectively. Here the ω ratios were estimated by the program, which make the model fit the data best (see Table S2-S4). LRTs for M1 and M3 pair can test whether the ω ratios of sites are significant different or not. The M1 model (neutral) assumes two classes of sites in proteins:the conserved sites (ω= 0) and the neutral sites (ω= 1). The M2 model (selection) adds a third class of sites with ω as a free parameter, thus allowing for sites with x > 1. The M7 model (beta) allows sites to have 10 different ω ratios in the interval (0, 1), which are calculated from the beta distribution with parameters p and q. Model M8 (beta and ω) adds an extra class of sites to the beta (M7) model and allows the sites to have ω> 1. LRTs for M1/M2 and M7/M8 will indicate whether the positive Darwin selection occurs or not in sequence level. Regarding the parameter-rich models (M7 and M8), the calculations were run twice as recommended by the author (<http://abacus.gene.ucl.ac.uk/software/> paml.html). Once with initial ω> 1 and again with ω< 1. The results corresponding to the higher likelihood value are used.

After ML estimates of parameters were obtained, the empirical Bayesian approach is used to calculate the probability that a specific site belongs to a given rate class depending on the data at that site (16,17). Anisimova et al. have indicated that the power and accuracy of the Bayesian prediction are particularly influenced by the divergent degree and numbers of the sequences analyzed. To obtain a reliable result, the authors recommended using multiple models to identify sites under positive selection(18). Sites with a high probability (>=90%) of coming from the class with ω> 1 are likely to be under positive selection and mapped onto structures by using a house Pymol command code generator.

**References** :

*15. Yang, Z. (1997) Comput Appl Biosci* ***13****(5), 555-556*

*16. Nielsen, R., and Yang, Z. (1998) Genetics* ***148****(3), 929-936*

*17. Yang, Z., and Bielawski, J. P. (2000) Trends in Ecology and Evolution* ***15****(12), 496-503*

*18. Anisimova, M., Bielawski, J. P., and Yang, Z. (2001) Molecular biology and evolution* ***18****(8), 1585-1592*
