# Supplementary figures and images for: Discovery of a Distinct Superfamily of Kunitz-Type Toxin (KTT) from Tarantulas
Source: PLoS One. 2008 Oct 15;3(10):e3414. doi: 10.1371/journal.pone.0003414 (PMC2561067; doi:10.1371/journal.pone.0003414)

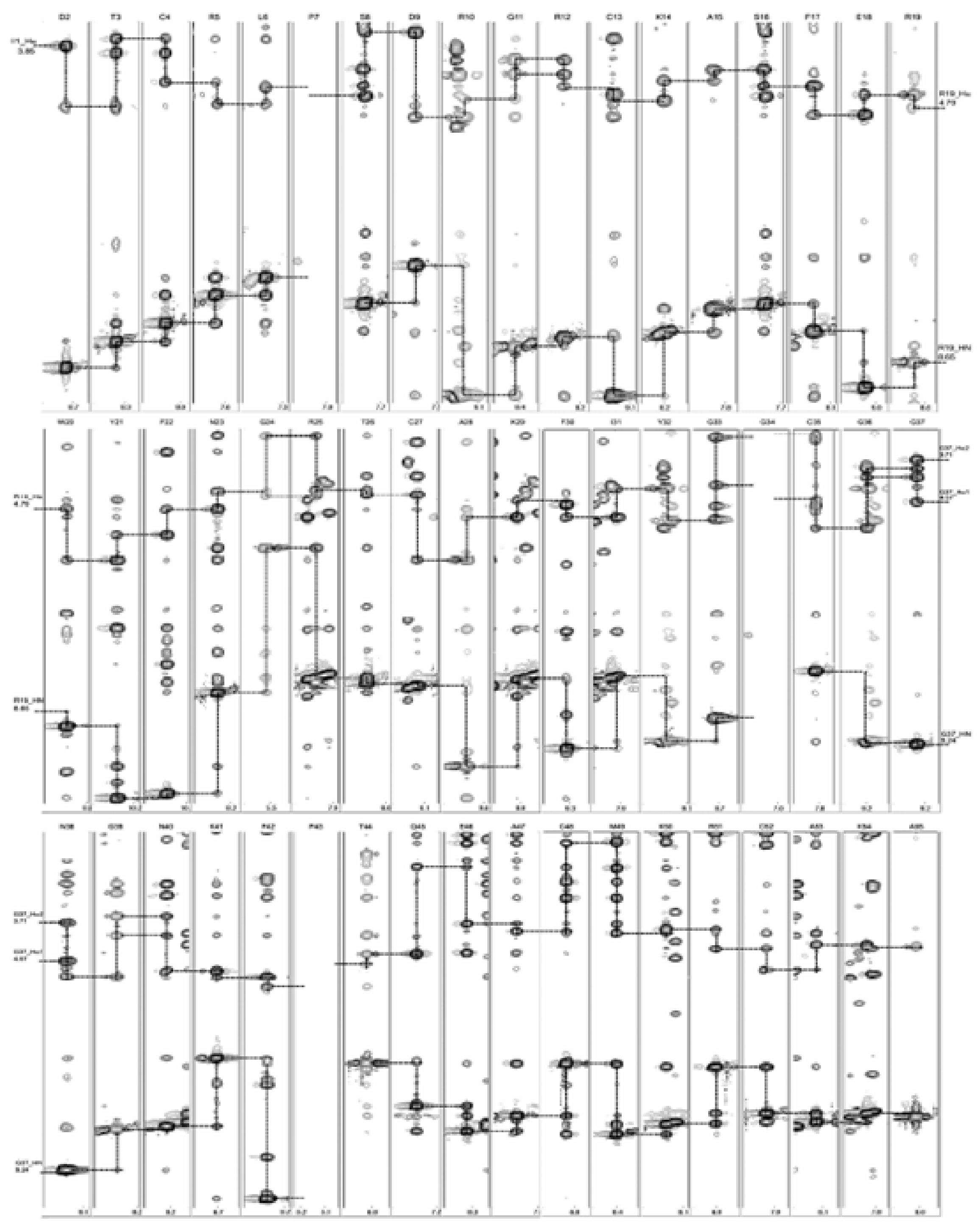

Supplement: Figure S1 — Sequential assignment of HWTX-XI by 3D 15N-1H NOESY-HSQC. Nearly complete dαN and dNN sequential connectivity was established throughout the sequence of HWTX-XI except for I1, P7, G34 and P43. (2.87 MB TIF) [file pone.0003414.s006.tif]

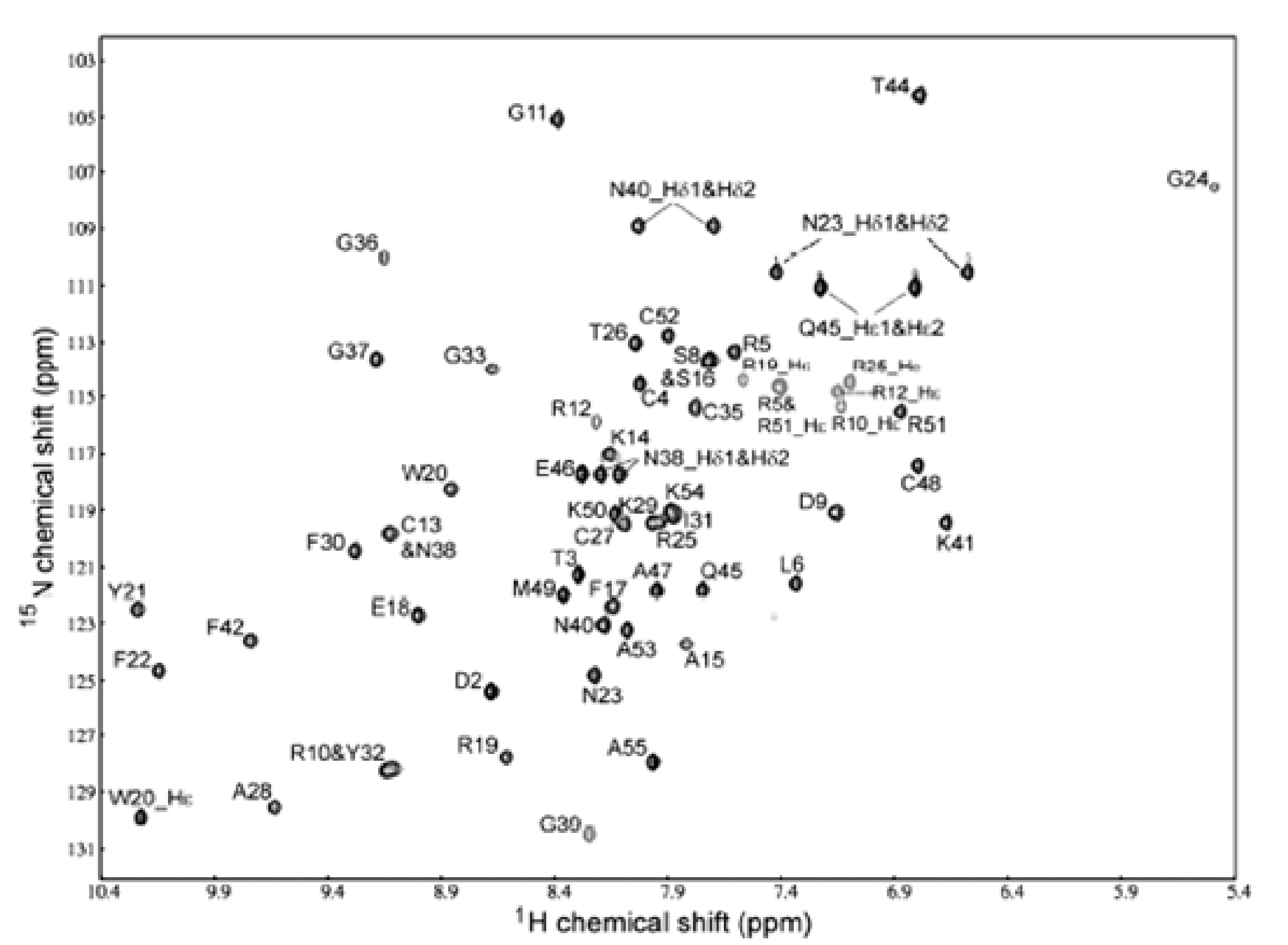

Supplement: Figure S2 — 2D 15N-1H HSQC spectrum of HWTX-XI. Amide NH assignments are annotated with the one-letter amino acid code and the sequence number, assignments of side-chain NH and NH2 groups are also shown. HN positions are shown by the dashed lines. (0.72 MB TIF) [file pone.0003414.s007.tif]

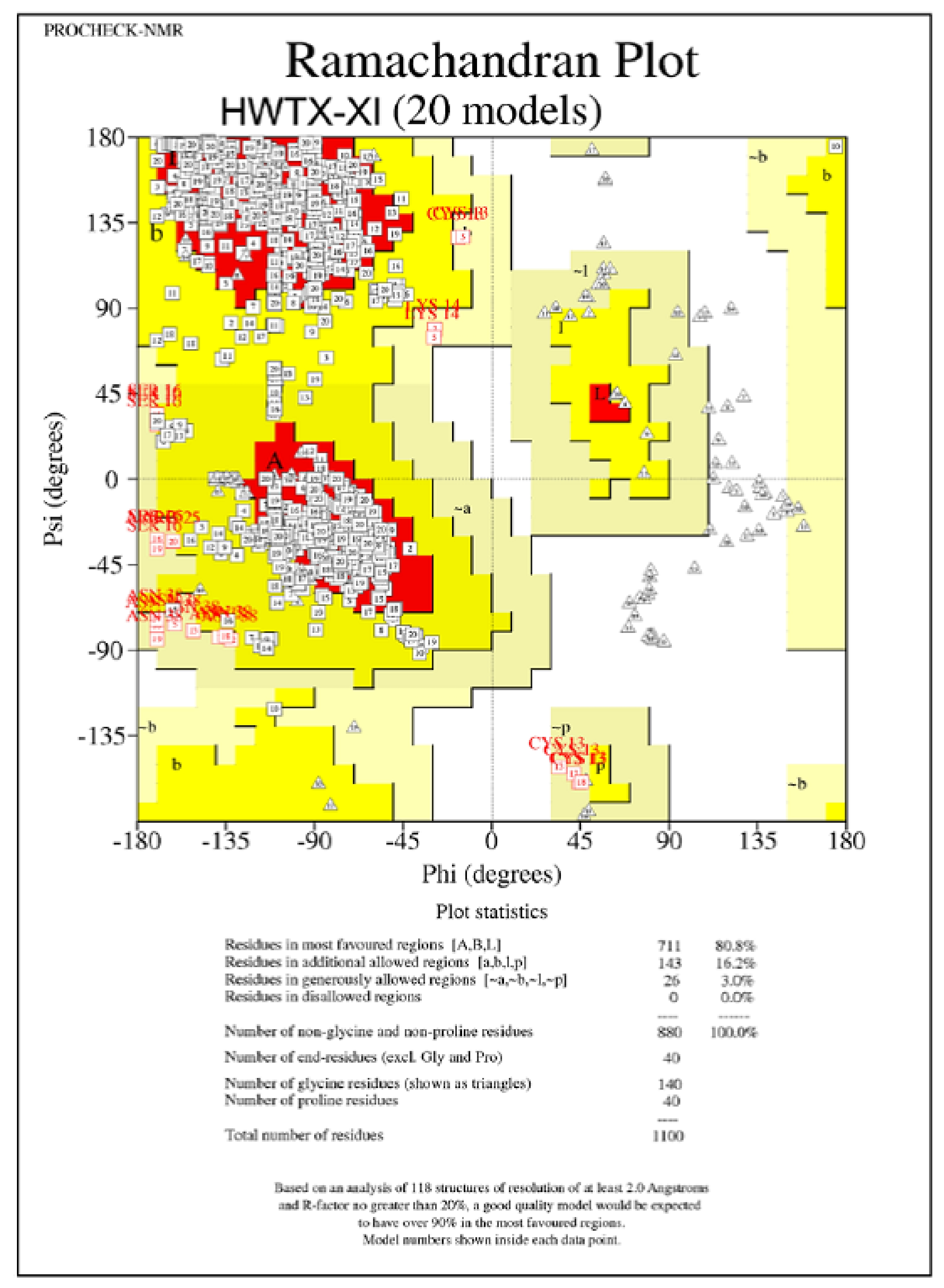

Supplement: Figure S3 — Analysis of the family of 20 structures using the program PROCHECK (3.60 MB TIF) [file pone.0003414.s008.tif]
