# Supplementary material for: Discovery of a Distinct Superfamily of Kunitz-Type Toxin (KTT) from Tarantulas
Source: PLoS One. 2008 Oct 15;3(10):e3414. doi: 10.1371/journal.pone.0003414 (PMC2561067; doi:10.1371/journal.pone.0003414)
Supplement: Table S1 — Chemical shifts of the assigned protons of HWTX-XI. (0.11 MB DOC) [file pone.0003414.s009.doc]

***Table S1:* Chemical shifts of the assigned protons of HWTX-XI.**

| **Residue** | **HN** | **15N** | **Hα** | **Hβ** | **Hγ** | **Hδ** | **Hε** | **Hζ** |
| --- | --- | --- | --- | --- | --- | --- | --- | --- |
| I1 |  |  | 3.85 | 1.94 | 1.53,1.24;  0.93# | 0.97# |  |  |
| D2 | 8.73 | 125.39 | 4.77 | 2.76# |  |  |  |  |
| T3 | 8.34 | 121.31 | 3.73 | 3.96 | 1.12# |  |  |  |
| C4 | 8.07 | 114.51 | 4.38 | 2.95# |  |  |  |  |
| R5 | 7.64 | 113.28 | 4.7 | 2.19,1.73 | 1.62# | 3.29# | 7.44 |  |
| L6 | 7.38 | 121.59 | 4.48 | 1.88,1.58 | 1.91 | 1.01,0.93 |  |  |
| P7 |  |  | 4.59 | 2.27,1.95 | 2.06,1.84 | 3.78,3.56 |  |  |
| S8 | 7.76 | 113.64 | 3.63 | 1.90,1.51 |  |  |  |  |
| D9 | 7.19 | 119.02 | 4.93 | 2.72,2.40 |  |  |  |  |
| R10 | 9.15 | 110.15 | 4.65 | 2.21,2.06 | 1.85# | 3.22# | 7.18 |  |
| G11 | 8.42 | 123.01 | 4.29,4.06 |  |  |  |  |  |
| R12 | 8.27 | 115.82 | 4.45 | 1.97,1.81 | 1.68,1.64 | 3.2# | 7.18 |  |
| C13 | 9.17 | 119.83 | 4.65 | 3.36,2.88 |  |  |  |  |
| K14 | 8.2 | 116.98 | 4.36 | 2.08,1.60 | 1.40,1.30 | 1.60# | 2.96# | 7.46 |
| A15 | 7.85 | 123.69 | 4.2 | 0.84# |  |  |  |  |
| S16 | 7.77 | 113.69 | 4.45 | 3.62,3.50 |  |  |  |  |
| F17 | 8.18 | 122.36 | 4.89 | 3.06# |  | 7.07# | 7.32# |  |
| E18 | 9.05 | 122.65 | 4.56 | 2.15,1.85 | 2.36,2.01 |  |  |  |
| R19 | 8.65 | 109.79 | 4.79 | 1.73,0.98 | 1.05,1.29 | 3.61,2.84 | 7.61 |  |
| W20 | 8.89 | 118.2 | 5.76 | 3.07,2.84 | Hδ1=6.75 | Hε1=10.27, Hε3=7.01; |  | Hζ3=7.07;  Hζ2=7.52;  Hη2=7.21 |
| Y21 | 10.28 | 122.48 | 5.29 | 2.95,2.79 | 7.06# | 6.68# |  |  |
| F22 | 10.19 | 124.62 | 4.81 | 3.70,2.90 | 7.51# | 7.21# | 7.29 |  |
| N23 | 8.26 | 124.77 | 4.43 | 3.38,1.97 | 7.47,6.62 |  |  |  |
| G24 | 5.52 | 125.52 | 4.54.3.39 |  |  |  |  |  |
| R25 | 7.97 | 119.38 | 4.39 | 1.76,1.64 | 1.52# | 3.16# | 7.14 |  |
| T26 | 8.08 | 113.02 | 4.51 | 4.14 | 1.04# |  |  |  |
| C27 | 8.13 | 119.46 | 5.76 | 3.51,2.88 |  |  |  |  |
| A28 | 9.68 | 111.53 | 4.95 | 1.33# |  |  |  |  |
| K29 | 8.02 | 119.41 | 4.63 | 1.16# | 0.11,-0.16 | 1.26# | 2.77, 2.71 | 7.47 |
| F30 | 9.32 | 120.37 | 4.87 | 3.01,2.90 | 7.06# | 7.18# |  |  |
| I31 | 7.92 | 119.13 | 4.29 | 1.75 | 1.43&1.18,0.71# | 0.64# |  |  |
| Y32 | 9.18 | 110.22 | 4.91 | 2.48,2.41 |  |  |  |  |
| G33 | 8.72 | 113.95 | 4.23,3.31 |  |  |  |  |  |
| G34 | 7.71 | 108.87 | 4.51,3.23 |  |  |  |  |  |
| C35 | 7.82 | 115.35 | 5.08 | 3.84,2.98 |  |  |  |  |
| G36 | 9.2 | 109.97 | 4.07,3.91 |  |  |  |  |  |
| G37 | 9.24 | 113.24 | 4.57,3.71 |  |  |  |  |  |
| N38 | 9.17 | 119.83 | 4.92 | 3.10,2.88 | 8.24,8.16 |  |  |  |
| G39 | 9.29 | 119.41 | 4.02,3.59 |  |  |  |  |  |
| N40 | 8.22 | 123.02 | 4.81 | 3.15,2.89 | 8.07,7.74 |  |  |  |
| K41 | 6.71 | 119.44 | 4.94 | 1.68,1.49 | 1.09,0.99 | 1.22,0.50 | 2.95, 2.62 | 7.09 |
| F42 | 9.78 | 123.63 | 5.15 | 3.19,2.74 | 7.29# | 6.82# |  |  |
| P43 |  |  | 4.63 | 2.46,2.25 | 2.33,2.20 | 4.05,4.00 |  |  |
| T44 | 6.83 | 122.22 | 4.4 | 4.4 | 1.23# |  |  |  |
| Q45 | 7.79 | 121.79 | 2.53 | 1.17.-0.54 | 1.81,1.61 | 7.28,6.86 |  |  |
| E46 | 8.33 | 117.69 | 3.76 | 1.99,1.79 | 2.39,2.36 |  |  |  |
| A47 | 8 | 121.83 | 3.91 | 1.55# |  |  |  |  |
| C48 | 6.84 | 117.45 | 1.99 | 3.22,2.85 |  |  |  |  |
| M49 | 8.4 | 121.98 | 3.97 | 1.96,1.91 | 2.54,2.06 | 0.63# |  |  |
| K50 | 8.17 | 119.17 | 3.87 | 1.75,1.71 | 1.61# | 1.47,1.37 | 2.93# | 7.48 |
| R51 | 6.9 | 115.49 | 4.31 | 1.91,1.77 | 1.72,1.63 | 3.47,3.28 | 7.45 |  |
| C52 | 7.94 | 112.75 | 4.78 | 2.02,1.79 |  |  |  |  |
| A53 | 8.13 | 123.22 | 4.21 | 1.48# |  |  |  |  |
| A54 | 7.94 | 119.03 | 4.28 | 1.85,1.72 | 1.38# | 1.66# | 2.98# | 7.49 |
| A55 | 8.02 | 109.98 | 4.2 | 1.33# |  |  |  |  |
