# Supplementary material for: Discovery of a Distinct Superfamily of Kunitz-Type Toxin (KTT) from Tarantulas
Source: PLoS One. 2008 Oct 15;3(10):e3414. doi: 10.1371/journal.pone.0003414 (PMC2561067; doi:10.1371/journal.pone.0003414)
Supplement: Table S2 — Parameter estimates and likelihood ratio statistics (2△l) for the BPTI like body proteins (0.03 MB DOC) [file pone.0003414.s010.doc]

Table S2. Parameter estimates and likelihood ratio statistics (2△l) for the BPTI like body proteins

| **Model** | **l** | **Estimates of parameters** | **2△l** | **Positively selected sites** |
| --- | --- | --- | --- | --- |
| **M0 (one ratio)** | -2336.64 | ω= 0.13131 |  | None |
| **M3 (discrete)** | -2187.48 | P_0_=0.260, ω_0_=0.005  P_1_=0.207, ω_1_=0.086  P_2_=0.532, ω_2_=0.362 | 298.32(13.28) | - |
| **M1 (neutral)** | -2222.39 | P_0_=0.399, ω_0_=0.046  P_1_=0.600, ω_1_=1.000 |  | Not allowed |
| **M2 (selection)** | -2222.39 | P_0_=0.399, ω_0_=0.046  P_1_=0.370, ω_1_=1.000  P_2_=0.230, ω_2_=1.000 | 0(9.21) | - |
| **M7 (β)** | -2185.52 | P=0.437, q=1.378 |  | Not allowed |
| **M8 (β& ω)** | -2183.82 | P_1_=0.033, ω=4.269  P_0_= 0.968  P=0.468, q=1.696 | 4(9.21) | M15 |

*Note*: Numbers in parentheses represent the critical values of Χ21% with df = 4 (M0/M3) or 2 (M1/M2, M7/M8). Positively selected sites are those with posterior probabilities (p) > 0.90, and those with p > 0.95 are in bold face by Naive Empirical Bayes (NEB) analysis.
