# Supplementary material for: Discovery of a Distinct Superfamily of Kunitz-Type Toxin (KTT) from Tarantulas
Source: PLoS One. 2008 Oct 15;3(10):e3414. doi: 10.1371/journal.pone.0003414 (PMC2561067; doi:10.1371/journal.pone.0003414)
Supplement: Table S3 — Parameter estimates and likelihood ratio statistics (2△l) for the snake KTTs (0.04 MB DOC) [file pone.0003414.s011.doc]

Table S3. Parameter estimates and likelihood ratio statistics (2△l) for the snake KTTs

| **Model** | **l** | **Estimates of parameters** | **2△l** | **Positively selected sites** |
| --- | --- | --- | --- | --- |
| **M0 (one ratio)** | -1783.64 | ω=1.169 |  | All residues |
| **M3 (discrete)** | -1705.32 | P_0_=0.260, ω_0_=0.098  P_1_=0.488, ω_1_=1.339  P_2_=0.250, ω_2_=5.826 | 156.64(13.28) | **A1, K3, Y4, K6,** L7, P8, **L9, R10, I11, G12, P13, K15, R16, K17, I18, P19, S20, K24, W25, K26, A27, K28, Q29, L31, P32, D34, S36, G39, A42, K46, I48, E49, E50, R52, R53, T54, V56, G57** |
| **M1 (neutral)** | -1756.37 | P_0_=0.348, ω_0_=0.088  P_1_=0.652, ω_1_=1.000 |  | Not allowed |
| **M2 (selection)** | -1706.54 | P_0_=0.249, ω_0_=0.074  P_1_=0.461, ω_1_=1.000  P_2_=0.289, ω_2_=5.138 | 99.6(9.21) | **K3, I11, P13, K15, K17, I18, W25, K26, A27, Q29, P32, D34,** R52 |
| **M7 (β)** | -1754.83 | P=0.437, q=1.378 |  | Not allowed |
| **M8 (β& ω)** | -1707.64 | P_1_=0.033, ω=4.269  P_0_= 0.968  P=0.468, q=1.696 | 94(9.21) | **K3, I11, P13, K15,** R16, **K17, I18, W25, K26, A27, Q29, P32, D34,** **G39**, R52 |

*Note*: Numbers in parentheses represent the critical values of Χ21% with df = 4 (M0/M3) or 2 (M1/M2, M7/M8). Positively selected sites are those with posterior probabilities (p) > 0.90, and those with p > 0.95 are in bold face by Empirical Empirical Bayes (EEB) analysis. The protein P00981 used as reference.
