# Supplementary material for: Discovery of a Distinct Superfamily of Kunitz-Type Toxin (KTT) from Tarantulas
Source: PLoS One. 2008 Oct 15;3(10):e3414. doi: 10.1371/journal.pone.0003414 (PMC2561067; doi:10.1371/journal.pone.0003414)
Supplement: Table S4 — Parameter estimates and likelihood ratio statistics (2△l) for the spider KTTs (0.03 MB DOC) [file pone.0003414.s012.doc]

Table S4. Parameter estimates and likelihood ratio statistics (2△l) for the spider KTTs

| **Model** | **l** | **Estimates of parameters** | **2△l** | **Positively selected sites** |
| --- | --- | --- | --- | --- |
| **M0 (one ratio)** | -529.56 | ω= 2.32358 |  | All residues |
| **M3 (discrete)** | -529.39 | P_0_=0.260, ω_0_=0.005  P_1_=0.207, ω_1_=0.086  P_2_=0.532, ω_2_=0.362 | 0.34(13.28) | All residues |
| **M1 (neutral)** | -539.82 | P_0_=0.204, ω_0_=0.000  P_1_=0.796, ω_1_=1.000 |  | Not allowed |
| **M2 (selection)** | -529.56 | P_0_=0.546, ω_0_=1.000  P_1_=0.276, ω_1_=1.000  P_2_=0.179, ω_2_=8.256 | 20.6(9.21) | **R1**, I2, K15, **R20** |
| **M7 (β)** | -540.73 | P= 4.912, q= 0.005 |  | Not allowed |
| **M8 (β& ω)** | -529.56 | P_1_=0.179, ω=8.256  P_0_= 0.821  P= 0.960, q= 0.005 | 22.4(9.21) | **R1**, I2, K15, **R20** |

*Note*: Numbers in parentheses represent the critical values of Χ21% with df = 4 (M0/M3) or 2 (M1/M2, M7/M8). Positively selected sites are those with posterior probabilities (p) > 0.90, and those with p > 0.95 are in bold face by Empirical Empirical Bayes (EEB) analysis.
